# Molecular docking, pharmacokinetic studies, and in vivo pharmacological study of indole derivative 2-(5-methoxy-2-methyl-1H-indole-3-yl)-N′-[(E)-(3-nitrophenyl) methylidene] acetohydrazide as a promising chemoprotective agent against cisplatin induced organ damage

**DOI:** 10.1038/s41598-021-84748-y

**Published:** 2021-03-18

**Authors:** Suhail Razak, Tayyaba Afsar, Nousheen Bibi, Mahmoud Abulmeaty, Wajhul Qamar, Ali Almajwal, Anam Inam, Dara Al Disi, Maria Shabbir, Mashooq Ahmad Bhat

**Affiliations:** 1 Department of Community Health Sciences, College of Applied Medical Sciences, King Saud University, Riyadh, Kingdom of Saudi Arabia; 2 Department of Bioinformatics, Shaheed Benazir Bhutto Women University, Khyber Pakhtunkhwa, Peshawar, Pakistan; 3 Department of Pharmocology and Toxicology, Central Laboratory, College of Pharmacy, King Saud University, Riyadh, 11451 Kingdom of Saudi Arabia; 4 Department of Pharmaceutical Chemistry, College of Pharmacy, King Saud University, Riyadh, 11451 Kingdom of Saudi Arabia; 5 Atta-Ur-Rahman School of Applied Biosciences, National University of Sciences and Technology, Islamabad, Pakistan

**Keywords:** Cancer, Computational biology and bioinformatics, Molecular biology

## Abstract

Cisplatin is an efficient anticancer drug against various types of cancers however, its usage involves side effects. We investigated the mechanisms of action of indole derivative, 2-(5-methoxy-2-methyl-1H-indol-3-yl)-N'-[(E)-(3-nitrophenyl) methylidene] acetohydrazide (MMINA) against anticancer drug (cisplatin) induced organ damage using a rodent model. MMINA treatment reversed Cisplatin-induced NO and malondialdehyde (MDA) augmentation while boosted the activity of glutathione peroxidase (GPx), and superoxide dismutase (SOD). The animals were divided into five groups (*n* = 7). Group1: Control (Normal) group, Group 2: DMSO group, Group 3: cisplatin group, Group 4: cisplatin + MMINA group, Group 5: MMINA group. MMINA treatment normalized plasma levels of biochemical enzymes. We observed a significant decrease in CD4^+^COX-2, STAT3, and TNF-α cell population in whole blood after MMINA dosage. MMINA downregulated the expression of various signal transduction pathways regulating the genes involved in inflammation i.e. *NF-κB, STAT-3, IL-1, COX-2, iNOS, and TNF-α*. The protein expression of these regulatory factors was also downregulated in the liver, kidney, heart, and brain. In silico docking and dynamic simulations data were in agreement with the experimental findings. The physiochemical properties of MMINA predicted it as a good drug-like molecule and its mechanism of action is predictably through inhibition of ROS and inflammation.

## Introduction

Cisplatin, being the most widely used chemotherapeutic agent in the treatment of an assortment of tumors^1^. Regardless of its positive antineoplastic effects, cisplatin aftershoots undesirable toxicities including cardiotoxicity, hepatic-toxicity, nephrotoxicity, and ototoxicity^2^. The toxicities limit its application in clinical oncology as a powerful chemotherapeutic agent^3^. Nephrotoxicity and hepatotoxicity are dose-limiting side effects in cisplatin-based chemotherapy^4^. Cisplatin accrues in the liver at considerable amounts second to the kidney^5^. Several studies cleared that the generation of reactive oxygen species (ROS) such as superoxide anion and hydroxyl radical is involved in the mechanism of cisplatin toxicity^3^ which leads to an elevation in lipid peroxidation (LPO), reduction in the level of protein-bound sulfhydryl groups and glutathione^6^. Furthermore, apoptosis as well as pro-inflammatory genes, inducible cyclooxygenase enzyme (COX-2), and inducible nitric oxide synthase (iNOS) might play a vital part in the mechanism of cisplatin-induced acute liver damage^7,8^. Toll-like receptor-4 (TLR-4) is a crucial intermediary in the initiation of innate immunity that leads to immense production of pro-inflammatory cytokines, consequently initiating an inflammatory reaction and aggravating the liver injury^9^. Recently, TLR-4 has been implicated in cisplatin-induced nephrotoxicity, ototoxicity, and polyneuropathy^10^. So, it is deemed of great importance to investigate ways for preventing the dose-limiting side effects of cisplatin at its tumoricidal doses for safer clinical use.

Recent studies proposed a crucial connection of activator of transcription (STAT) family proteins especially STAT3 and signal transducers in selectively encouraging and sustaining a carcinogenic inflammatory microenvironment, equally at the beginning of malignant alteration and throughout cancer progression^11,12^. STAT3 is allied to inflammation linked tumorigenesis that is initiated by genetic variations in various malignancies^13,14^. STAT 3 has shown a strong relationship with nuclear factor-κB (NF-κB) signaling^15,16^. It is systematically significant that STAT3 and NF-κB interact with each other at numerous levels in an exceedingly context-dependent approach. Some inflammatory factors encoded by NF-κB target genes, furthermost particularly interleukin- 6 (IL-6), are significant STAT3 activators. NF-κB and STAT3 similarly co-regulate several inflammatory genes^17^.

Indomethacin belongs to non-steroid anti-inflammatory drugs (NSAID) and is the derivative of indole acetic acid, is known for causing gastric bleeding and gastric ulcers. Though, by chemical modifications, the profile of its safety has been improved^18^. Studies have illustrated that modification by synthesis increased the probability to provide derivatives with noteworthy anti-inflammatory and lessen the chance of side effects^19^. The solution for the above-mentioned criteria has been limited by the COX-2 inhibitors^20–22^. Indole is an imperative scaffold in the field of medicinal chemistry. Studies revealed that indole derivatives have shown vital pharmacological activities like anti-inflammatory, antipyretic, anticancer, and analgesic^23,24^. Schiff bases have been reported to possess various pharmacological activities e.g. anticonvulsant, anti-inflammatory activity^25^, and anti-tubercular^26^.

Hansch and Fujita^27^ has developed Quantitative structure–activity relationship (QSAR) as ligand-based drug design approach. To get a reliable statistical model for the prediction of activities of new chemical molecule QSAR has been used by years ago to develop relationships among physicochemical properties of chemical molecule and their biological activities. The principle underlying the QSAR modeling is that the variation among the biological properties of chemical compunds depend upon their structural properties^28^. The intramolecular interactions between the chemical compound and protein or protein and protein can be precited through molecular docking. Dicking modes of the resulting protein–ligand or protein–protein complexes are responsible for the inhibition of receptor molecule. Binding free energy and number of steric and hydriphbic interactions are responsible to fit the ligand molcelue into the cavity of receptor protein^29^. Nowadays it is quite evident that combination of molecular docking experimental highthroughput/clinical data is crucial in the process of drug discovery.

The present study aimed to investigate the possible protective effects of indole derivative, 2-(5-methoxy-2-methyl-1H-indol-3-yl)-N'-[(E)-(3-nitrophenyl) methylidene] acetohydrazide (MMINA) against cisplatin-induced hepatotoxicity, nephrotoxicity, cardiotoxicity, and neurotoxicity in rats. We have analyzed various enzymatic as well as molecular biomarkers to find out mechanisms of action of MMINAagainst cisplatin-induced toxicities. Histopathological studies were performed to study the changes at the morphological level. The physicochemical, biological, and environmental properties of the MMINA compound were examined from the knowledge of its chemical structure and QSAR. Furthermore, molecular docking studies were also performed to depict the molecular targets of MMINA against cisplatin organ damage.

## Results

**Figure 1:**
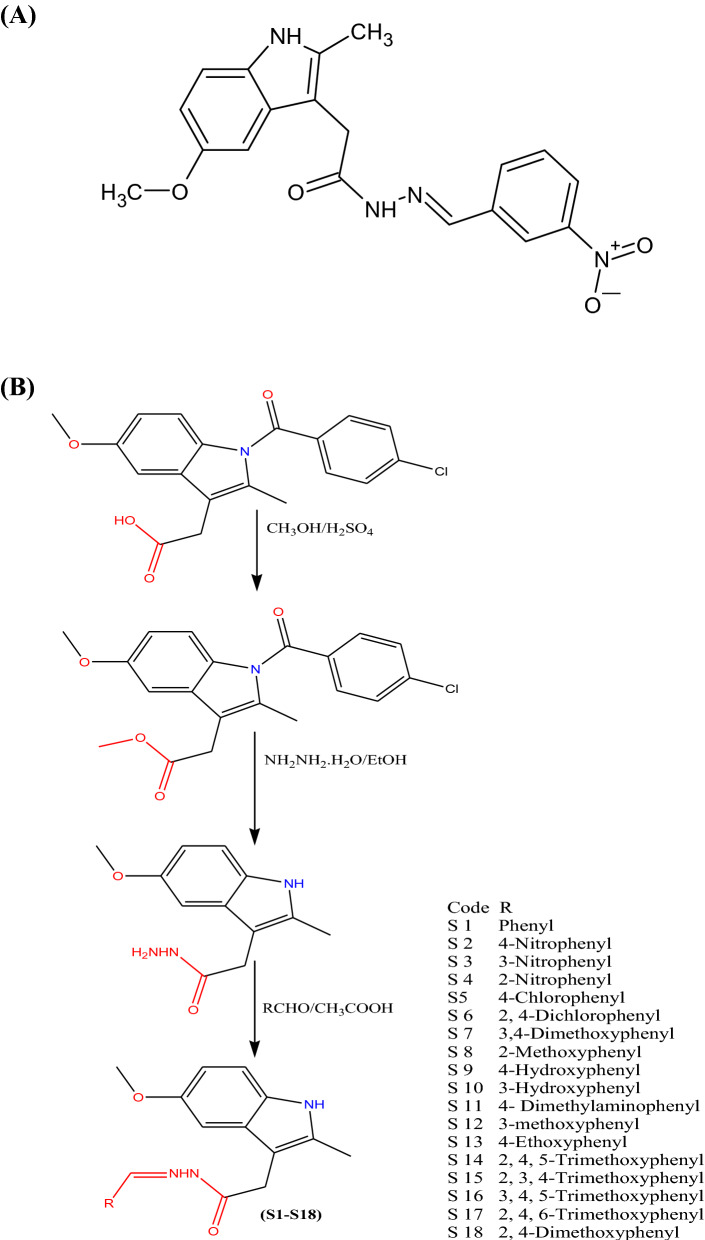
(**A**) Structure of a novel indole derivative, 2-(5-methoxy-2-methyl-1H-indol-3-yl)-N'-[(E)-(3- nitrophenyl) methylidene] acetohydrazide (MMINA). (**B**) Scheme 1.

The starting material, indole hydrazide, 2-(5-methoxy-2-methyl-1H-indol-3-yl) acetohydrazide (1) was used for the synthesis of MMINA, 2-(5-methoxy-2-methyl-1H-indole-3-yl)-N’-[(E)-(3-nitrophenyl) methylidene] acetohydrazide^30^. To obtain the desired hydrazone, indole hydrazide was refluxed with 3-nitrobenzaldehde in methanol, and the glacial acetic acid was used as a catalyst. An efficient synthetic route (Scheme 1)^31^ was used to achieve the synthesis of the compound MMINA (Supplementary file S2; Fig. 1). The structure of the compound was allocated by the ^1^H-NMR and ^13^C-NMR. Furthermore, elemental analysis, FT-IR (Perkin Elmer), and mass spectrometry were used to characterize the structure of the compound. 2-(5-methoxy-2-methyl-1H-indol-3-yl) acetohydrazide (1) and 2-(5-methoxy-2-methyl-1H-indole-3-yl)-N’-[(E)-(3-nitrophenyl) methylidene] acetohydrazide (MMINA) showed similar NMR splitting and δ-values (δ_H_ & δ_C_) with pattern of H-3, H-5, and H-6 aromatic protons and their ^13^C signals. The disappearance of –NH_2_ protons at 4.26 ppm in ^1^H-NMR analysis confirmed the structure of the compound. A mass spectrum was used to confirm the molecular weight of the compound.

### MMINA treatment ameliorated cisplatin induce alterations in liver, kidney, heart and brain functions

**Figure 2:**
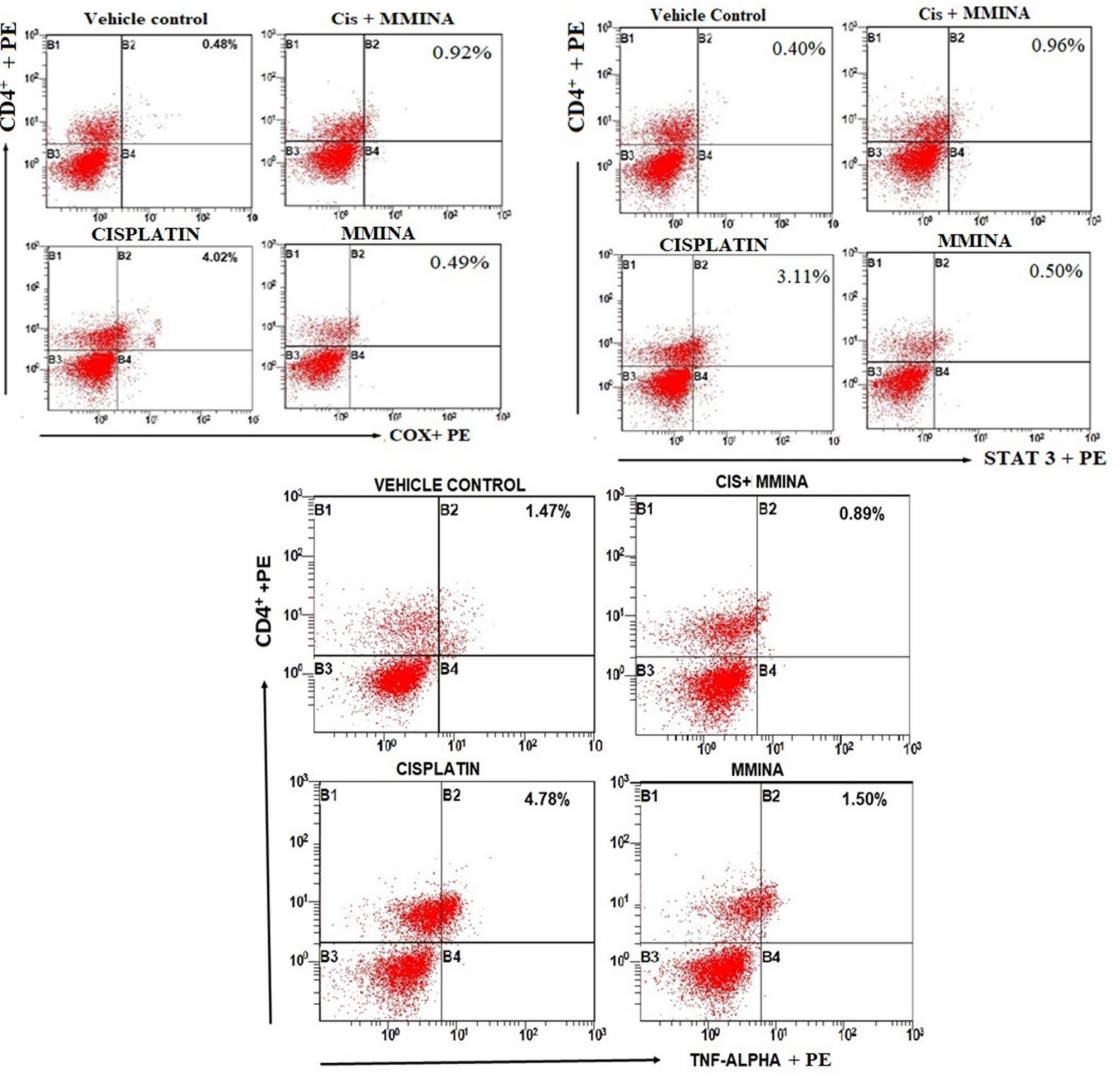
Effect of MMINA on CD4^+^COX2, CD4^+^STAT3, and CD4 + TNF-α population. Flow cytometric analysis of CD4^+^COX2, STAT3, TNF-α and CD4^+^ expressing cells in whole bllod from one rat from each group. Statistical analysis was performed using a one way ANOVA followed by the Tukey–Kramer post-test. Each value indicates the mean ± SEM of six animals.

The blood biochemistry results showed that creatinine (Cr) and urea levels were intriguingly (81 mg/dl and 85.5 ± mg/dl, *p* < 0.0001 respectively) altered upon single exposure of cisplatin (12 mg/kg, i.p) in rats. MMINA post-treatment after cisplatin exposure significantly restored the kidney functioning by lowing serum creatinine and urea levels to normal ranges (29.33 ± mg/dl and 36.67 ± mg/dl respectively) (Supplementary file S2; Fig. 2; I a and b). Liver injury is the main reason for high ALT, AST, and ALP levels in the serum. Results showed that ALT, AST, and ALP were considerably (*p* < 0.0001) raised in cisplatin exposed group (39.01 ± 0.85 IU/L, 157.1 ± 2.58 IU/L, and 154 ± 1.78 IU/L, respectively) compared with control groups (28.5 ± 0.62 IU/L, 21.66 ± 1.01 IU/L and 64.5 ± 1.72 IU/L, respectively) (Supplementary file S2; Fig. 2; II a, b, and c), whereas MMINA administration significantly restored the LFTs to their normal ranges. The normal levels of serum ALT, AST, and ALP following post-treatment of MMINA (25 mg/kg) for 7 days ensuing cisplatin inoculation ascertain the compound’s protective effect against cisplatin induce liver assault. MMINA (25 mg/kg) alone administration showed normal enzyme activity, but a slightly low range of enzymes that is comparable and insignificantly different from control group enzyme activity. The normal LFTs level stipulates normal functioning liver in the MMINA + cisplatin post-treatment group. CK-MB isoenzyme is the biomarker that appears in the blood after acute myocardial insult. We found that acute cisplatin exposure significantly raised CK-MB (65.17 ± 1.19 ng/ml) secretion in the blood indicative of myocardial injury. MMINA administration after cisplatin inoculation significantly reduced the release of CK-MB (24.33 ± 1.17 ng/ml) from the myocardium (Supplementary file S2; Fig. 2; III). Cisplatin inoculation caused significant increase in AChE activity in brain of the treated rats (76.5 ± 1.48 mM/min/mg protein) whereas treatment with MMINA significantly (28.33 ± 0.98 mM/min/mg protein, *p* < 0.0001) prevented the adverse effect of Cisplatin as shown by the decrease in AChE action in brain tissue (Supplementary file S2; Fig. 2; IV).

### Effect of MMINA treatment on lipid profile

Triglycerides (TGAs), total cholesterol, low-density lipoprotein (LDL), and high-density lipoprotein (HDL)-cholesterol in plasma were analyzed (Supplimentary file S1: Table 2). Significant changes in the blood lipid profile (Total cholesterol, HDL, LDL and TGAs were noticed in the cisplatin inoculated group (120.50 ± 2.82 mg/dl, 11.18 ± 0.83 mg/dl, 117.17 ± 2.45 mg/dl and 101.89 ± 2.88 mg/dl respectively). The post-inoculation of IND (25 mg/kg) in cisplatin-treated rats for 7 days had a promising effect on lowering total cholesterol (76.54 ± 2.01), LDL (50.31 ± 0.70) and TGAs (79.99 ± 1.24), while a significant rise in HDL (30.17 ± 0.67) was noticed. No differences were observed in MMINA alone and control groups.

### Oxidative stress and antioxidants biomarkers

Cisplatin treatment results in a decrease in the activities of brush border membrane (BBM) and free radical scavenging enzymes and induces oxidative stress. Since cisplatin treatment is associated with oxidative stress, we have studied the effect of MMINA on tissue MDA and NO content. Cisplatin triggered a remarkable (*p* < 0.0001) increase in tissues MDA and NO levels (Table 1). Post-treatment with MMINA (25 mg/kg) prevented oxidative damage in cisplatin-intoxicated rats, with no change in oxidative biomarker enzyme activity in the liver, kidneys, heart, and brain of normal rats. We noticed a decline in glutathione and SOD activity in the liver, kidney, heart, and brain tissue of cisplatin-administered rats (Table 2). Cisplatin decreased tissue’s GPx and SOD levels, an effect prevented profoundly by post-treatment of MMINA compound (25 mg/kg). Rats treated with 25 mg/kg MMINA alone showed normal GSH and SOD enzyme levels in all observed organs, indicating a protective and safe effect of MMINA treatment.

**Table 1:** Effect of treatment with MMINA on oxidative stress in cisplatin-treated rat organs.

Treatments	MDA (ng/mg)				NO (ng/mg)			
	Liver	Kidney	Heart	Brain	Liver	Kidney	Heart	Brain
Control	45.3 ± 2.11	34.65 ± 3.12	25.65 ± 2.15	32.79 ± 2.65	25.3 ± 1.11	31.65 ± 1.02	27.65 ± 1.17	19.79 ± 0.92
DMSO Vehicle	45.98 ± 2.34	35.01 ± 3.98	25.98 ± 2.01	32. 27 ± 2.34	25.98 ± 1.34	31.01 ± 0.98	27.98 ± 1.01	19. 97 ± 0.74
Cisplatin (12.5 mg/kg)	145.54 ± 3.76****	158.97 ± 4.87****	119.76 ± 5.21****	98.87 ± 3.11****	112.54 ± 2.71****	126.97 ± 3.37****	99.76 ± 5.21****	78.87 ± 1.61****
Cisplatin + MMINA	56.87 ± 3.21*,^++++^	51.87 ± 3.11*,^++++^	49.43 ± 3.08*,^++++^	40.76 ± 1.98^++++^	36.67 ± 2.21*,^++++^	40.87 ± 2.30^++++^	34.43 ± 1.28^++++^	29.06 ± 0.99*^,++++^
MMINA (25 mg/kg)	42.12 ± 2.87^++++^	32.78 ± 2.54^++++^	23.76 ± 1.97^++++^	30.23 ± 1.76^++++^	22.12 ± 1.27^++++^	30.18 ± 0.99^++++^	25.76 ± 1.07^++++^	18.23 ± 0.76 ^++++^

Data are mean ± SEM, (n = 6). *, ****p < 0.05 and *p* < 0.0001 versus Control respectively, and ^++++^*p* < 0.0001 versus CP. Data analyzed by One-way ANOVA followed by Tukey’s multiple comparison tests.

**Table 2:** Effect of treatment with MMINA on antioxidant parameters in cisplatin-treated rat organs.

Treatments	SOD (U/g)				GPx (nM/min/g)			
	Liver	Kidney	Heart	Brain	Liver	Kidney	Heart	Brain
Control	35.91 ± 0.47	46.67 ± 1.17	33.94 ± 0.94	29.96 ± 0.52	45.68 ± 1.09	55.25 ± 0.87	39.63 ± 0.92	41.52 ± 0.63
DMSO Vehicle	35.83 ± 0.81	46.35 ± 1.01	33.78 ± 0.96	30.04 ± 0.60	45.43 ± 0.99	54.97 ± 0.74	39.49 ± 0.63	41.24 ± 0.70
Cisplatin (12.5 mg/kg)	7.3 ± 0.65****	5.06 ± 0.46****	6.07 ± 0.61****	4.24 ± 0.03****	9.14 ± 0.25****	7.45 ± 0.54****	6.79 ± 0.79****	6.07 ± 0.60****
Cisplatin + MMINA	30.39 ± 0.97^++++^	39.87 ± 1.03^++++^	28.31 ± 0.77^++++^	23. 20 ± 0.57^++++^	39.32 ± 0.31^++++^	47.07 ± 0.55^++++^	31.27 ± 0.92^++++^	38.04 ± 0.65^++++^
MMINA (25 mg/kg)	38.66 ± 0.52^++++^	47.89 ± 1.13^++++^	34.60 ± 0.90^++++^	32.11 ± 0.96^++++^	46.01 ± 0.85^++++^	56.35 ± 0.67^++++^	41.17 ± 0.92^++++^	42.14 ± 0.81^++++^

Data are mean ± SEM, (n = 6). *, ****p < 0.05 and *p* < 0.0001 versus Control respectively, and ^++++^*p* < 0.0001 versus CP. Data analyzed by One-way ANOVA followed by Tukey’s multiple comparison tests.

### Flow cytometry analysis on the effect of MMINA on CD4^+^COX-2, CD4^+^STAT3, and CD4^+^TNF-α

We evaluated the effect of MMINA on the configuration of the T-cell population by flow cytometry. There was a substantial increase in the percentage of CD4^+^COX-2, CD4^+^STAT3, and CD4^+^TNF-α (Fig. 2) in the cisplatin group when compared to the vehicle control group. Prominently, following treatment with MMINA, there was a marked decrease in the percentage of CD4^+^COX-2, CD4^+^STAT3, and CD4^+^TNF-α cells when compared with the cisplatin group. These results proposed that the anti-inflammatory action shown by the MMINA in the cisplatin + MMINA group may be attributed to the reduction of COX-2, STAT3, and TNF-α.

### Quantification of mRNA expression of inflammatory mediators (IL-1, COX-2, and iNOS) in various tissues

**Figure 3:**
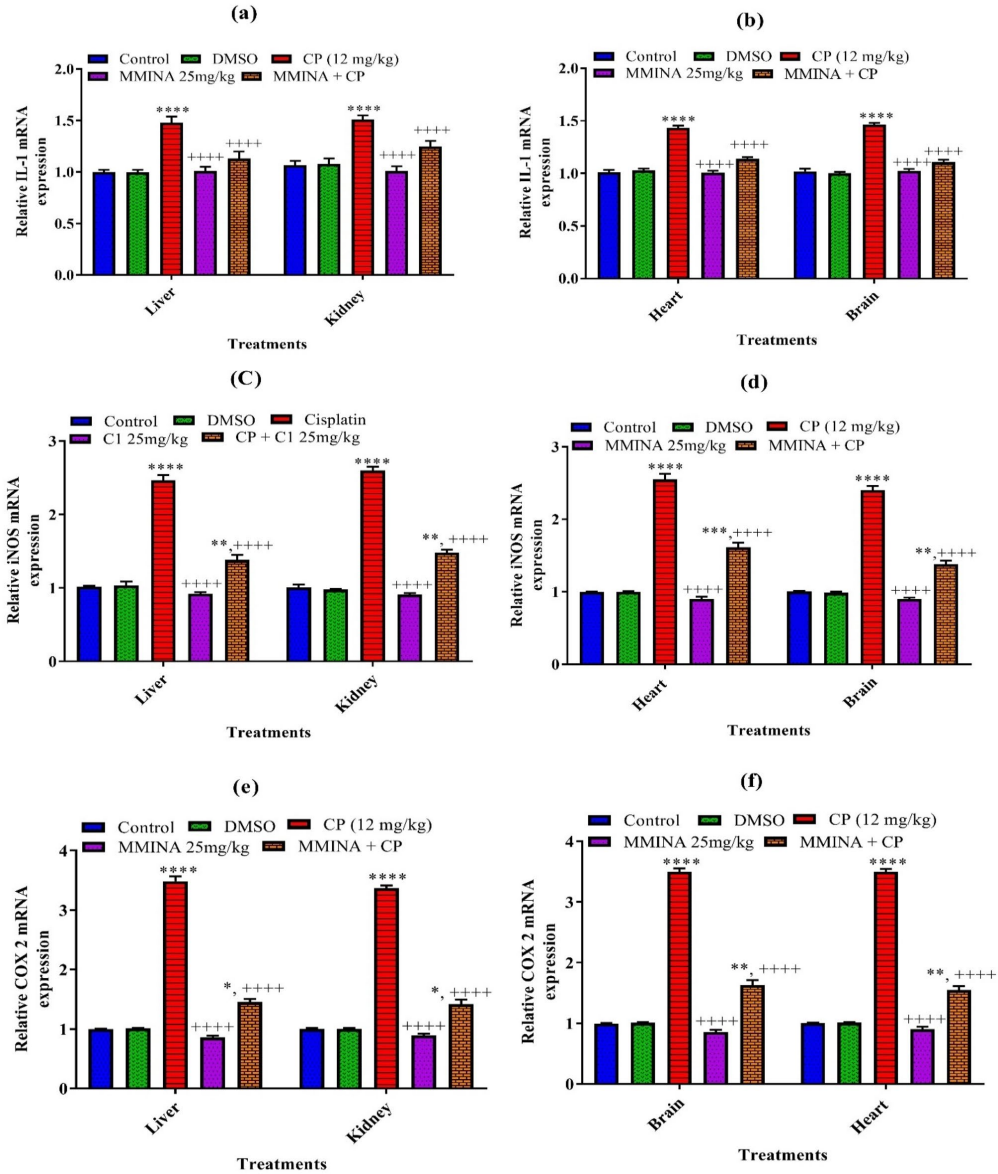
Effect of MMINA treatment on the gene expression of STAT-3, NF-κBp65, TNF-α. mRNA expression was measured by quantitative RT-PCR in the in Liver Kidney, heart and brain tissues. Data are mean ± SEM, (n = 6). *, ****p < 0.05 and *p* < 0.0001 versus Control respectively, and ^++++^*p* < 0.0001 versus CP. Data analyzed by One-way ANOVA followed by Tukey’s multiple comparison tests.

Our results revealed that there is a significant increase in the mRNA expression of inflammatory markers; IL-1, COX-2, and iNOS in the liver and kidney of cisplatin-treated animals as compared to the control group (Fig. 3). These responses were markedly attenuated by treatment with 25 mg/kg MMINA when compared with cisplatin (12 mg/kg) treated group in liver and kidney tissues.

Similarly, cisplatin-induced neurotoxicity and cardiotoxicity were categorized by a noteworthy increase (*p* < 0.0.001) in mRNA expression of IL-1, COX-2, and iNOS in the cisplatin group as compared to the normal group in brain and heart tissues. However, treatment of cisplatin-treated rats with MMINA reduced the mRNA expression of IL-1, COX-2, and iNOS significantly in heart and brain tissues as compared to the cisplatin alone-treated group.

### RT-PCR analysis of genes involved in the regulation of inflammatory markers (STAT-3, NF-κB, TNF-α) in various tissues

**Figure 4:**
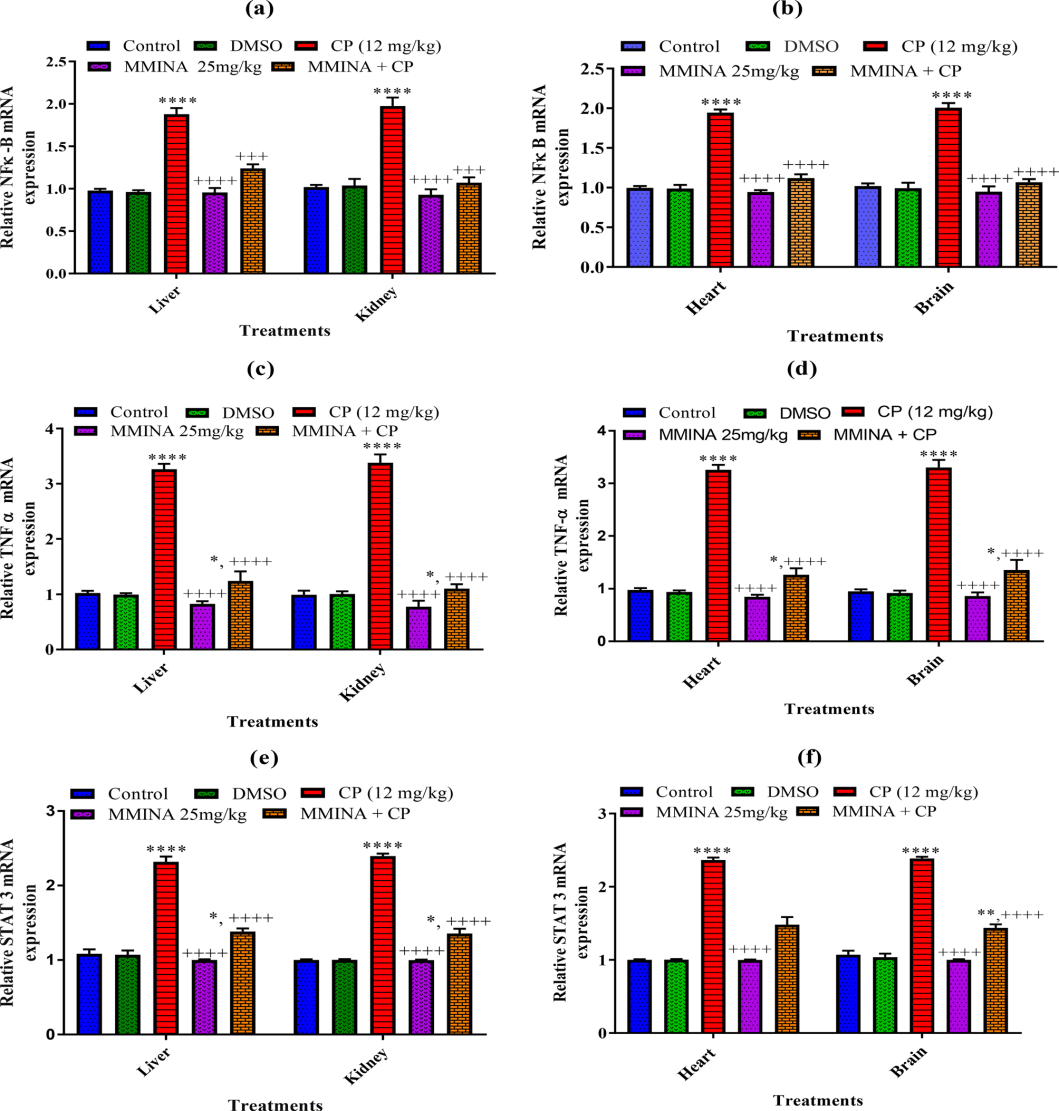
Effect of MMINA treatment on the gene expression of STAT-3, NF-κBp65, TNF-α. mRNA expression was measured by quantitative RT-PCR in the in Liver Kidney, heart and brain tissues. Data are mean ± SEM, (n = 6). *, ****p < 0.05 and *p* < 0.0001 versus Control respectively, and ^++++^*p* < 0.0001 versus CP. Data analyzed by One-way ANOVA followed by Tukey’s multiple comparison tests.

Inflammatory mediators like iNOS and COX-2 are transcriptionally controlled by NF-κB, which is retained in a sedentary state in resting through binding to Ikb. Our results documented by real-time PCR showed that cisplatin significantly increased the mRNA expression of the inflammatory mediators (STAT-3, NF-κBp65, TNF-α) in Liver and kidney tissues when compared to the control group (Fig. 4). Also, there was a significant decrease in mRNA expression of these regulatory genes in Liver and kidney tissues when compared with the cisplatin group.

Similarly, we observed that the gene expression of IL-1, STAT-3, NF-κBp65, TNF-α was significantly (*p* < 0.05) up-regulated in the cisplatin-treated group as compared to the control group (Fig. 4) in brain and heart tissues. The administration of MMINA demonstrated downregulation of STAT-3, NF-κB p65, and TNF-α significantly in the brain and heart tissue of rats when compared with the cisplatin-treated group.

### MMINA effect on protein expression of COX-2, STAT3, IL-1, TNF-alpha and NFкB p65 signal transducers

**Figure 5:**
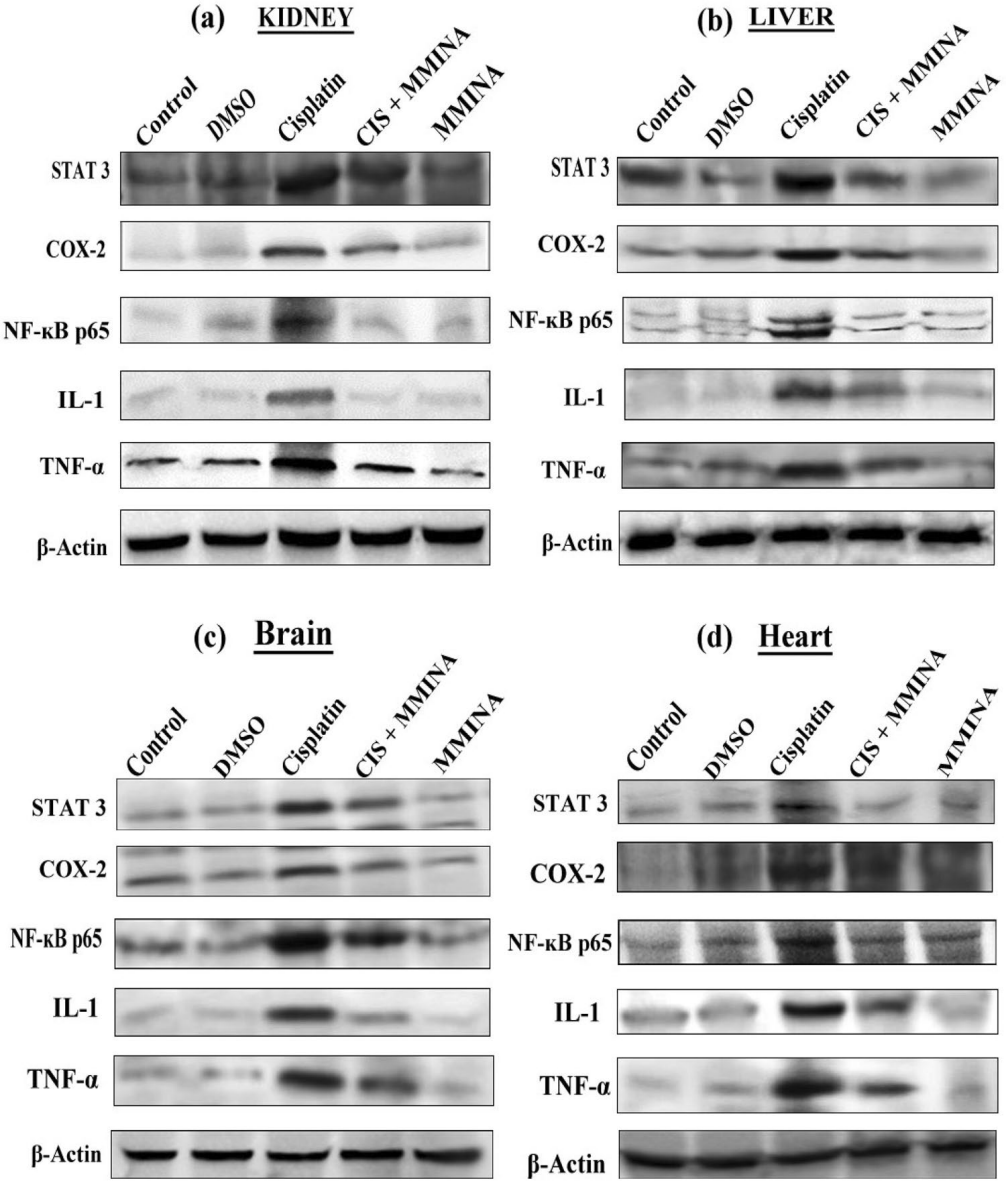
Western blot analysis of STAT3, COX2, NFĸB p65, IL1 and TNF-α protein expression in (**a**) Kidney, (**b**) Liver, (**c**) Brain and (**d**) Heart tissue lysates from different treatment groups. Treatments are indicated above the blots. Full sized blots are included in the Supplementary file S3. All gels and blots were run under the same experimental conditions as described in “Methods”.

Organ toxicities induced by cisplatin was categorized by a significant increase in liver, brain , heart and kidney tissue protein expression of TNF-α, COX-2, STAT3 IL-1 and NF-κB p65 of cisplatin group when compared with control group. Further, immunoblot analysis demonstrated that Cisplatin + MMINA treated group significantly downregulated liver, brain , heart and kidney tissue protein expression of TNF-α, COX-2, STAT3, IL-1 and NF-κB p65 (Fig. 5) when compared with cisplatin treated group.

### MMINA protects against cisplatin-induced injury in various organs

**Figure 6:**
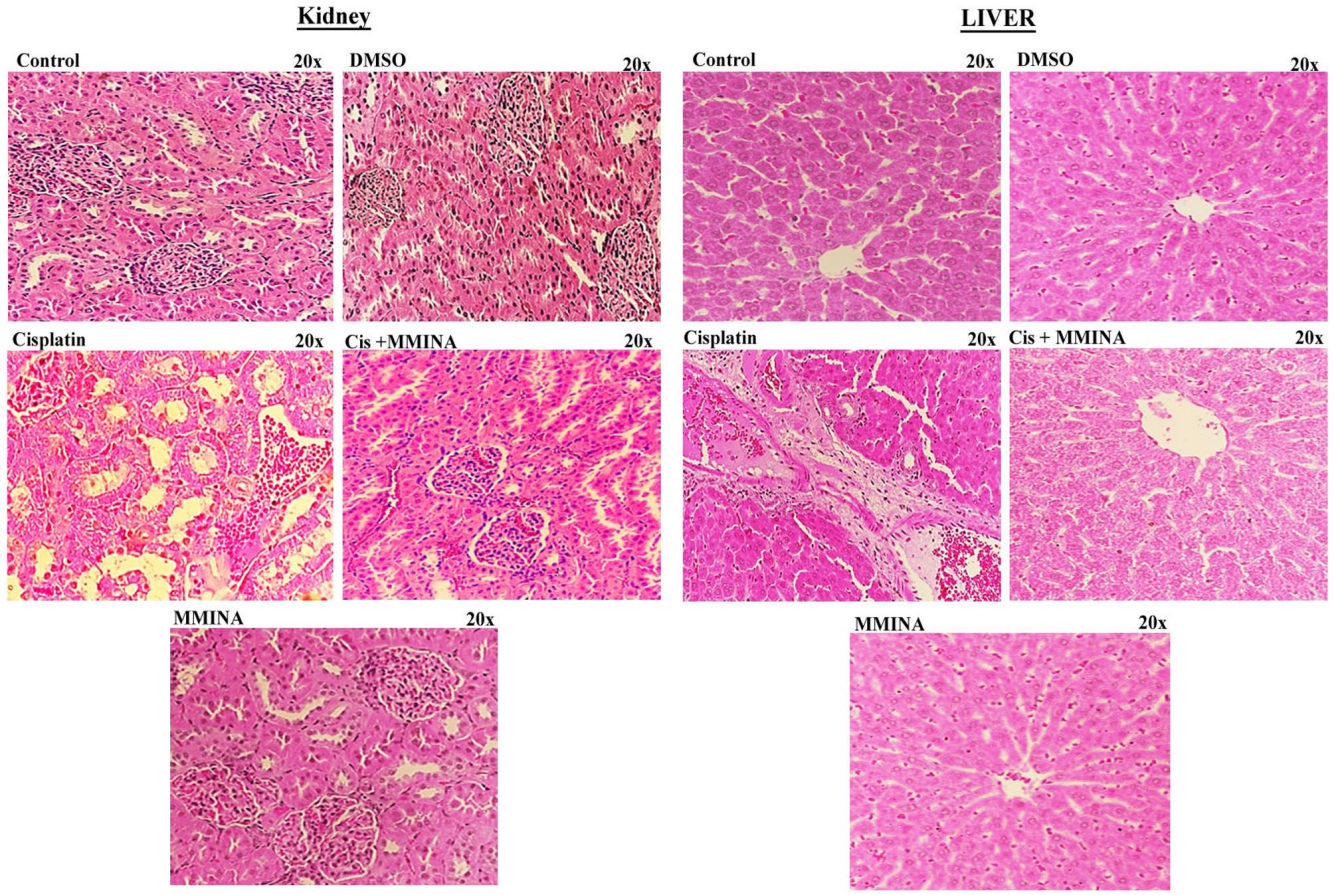
H & E staining of Liver and kidney tissues from various treatment groups at 20x.

Figure 6 depicts the photomicrograph of microscopic images of H & E stained liver and kidney tissues from various treatment groups. Histological examination and quantification revealed normal histology of rat kidneys (glomeruli structure encapsulated in Bowman’s capsule, tubules, interstitium, and blood vessels) in the control, DMSO (vehicle control), and MMINA alone treated groups. After inoculation of a single dose of cisplatin (12 mg/kg, i.p.), cisplatin-treated group exhibited severe renal damages, tubular necrosis, remarkable vacuolation, desquamation of epithelial cells in the renal tubules, dilatation of Bowman’s capsule, glomerular atrophy, pyknotic nuclei, proteinaceous casts in renal tubules, deterioration, necrosis, and detachment of the proximal tubular epithelial cell lining, shedding of the apical microvilli and lost cellular details. The damage was significantly attenuated by MMINA treatment in rats. The kidney of rats treated with MMINA showed mild tubular degenerations and the cellular structure appears normal with no proteinaceous cast observed. Morphological studies manifest that the renal tubule system is the site of maximum cisplatin damage while a shielding influence of MMINA was evident in the tubule system.

Examination of the H&E-stained sections showed normal liver architecture in control and MMINA-supplemented rats. The cisplatin-intoxicated rats showed degenerative changes, leukocyte infiltration, hemorrhage, cytoplasmic vacuolations, congestions, and other manifestations in hepatocytes. In contrast, rats received MMINA (25 mg /kg) after cisplatin injection exhibited remarkable amelioration of the liver histology with mild cytoplasmic vacuolations, leukocyte infiltration, and congestion.

**Figure 7:**
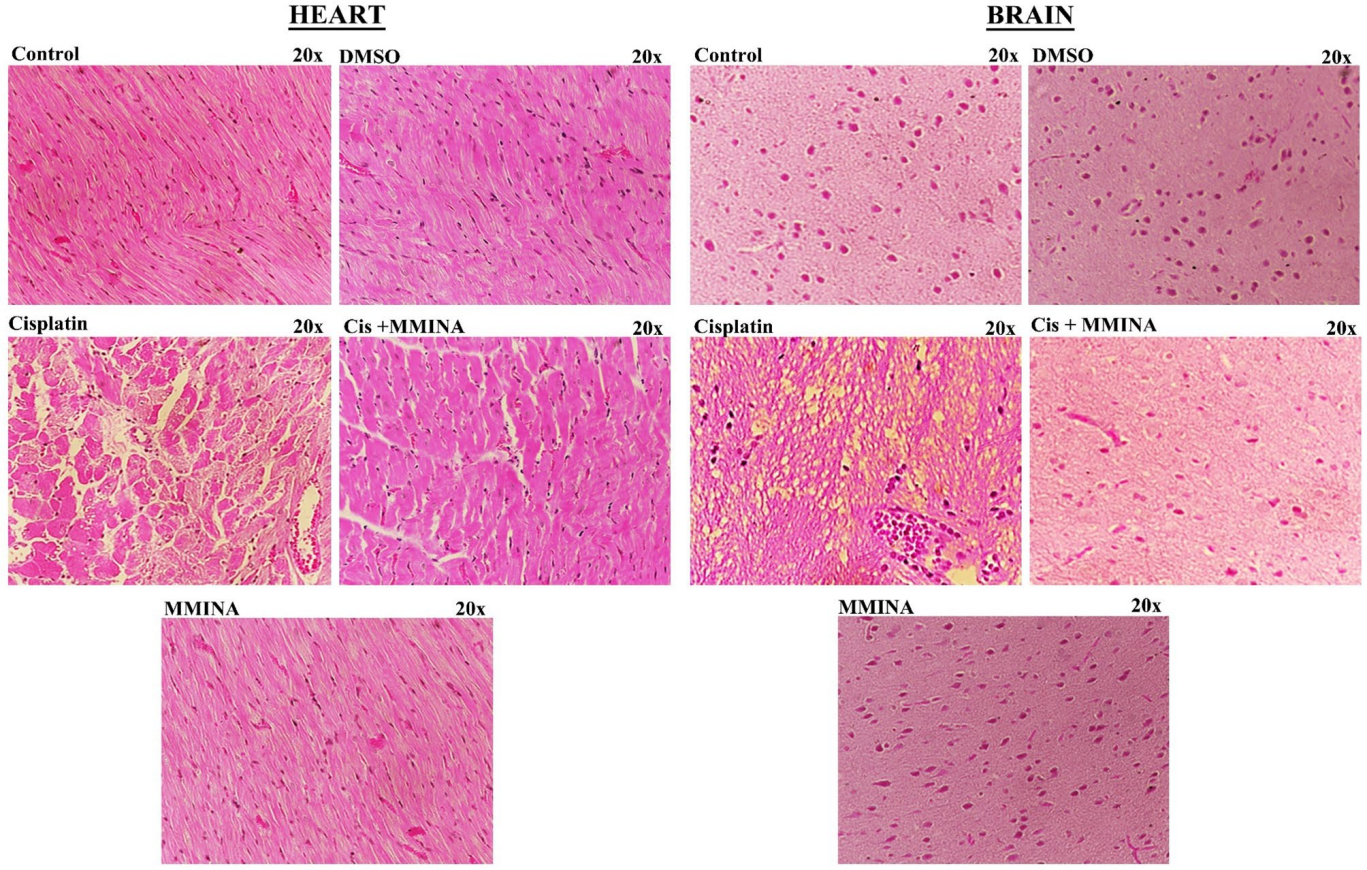
H & E staining of Heart and Brain tissues from various treatment groups at 20x.

Figure 7 depicts the photomicrograph of microscopic images of H &E stained heart and brain tissue sections from various treatment groups. The cardiac sections of control, DMSO, and MMINA treated groups indicated normal morphology of cardiac muscle fibers with several small blood vessels, myofibrillar structure with striations, branched appearances, and endurance with adjacent myofibrils and capillaries in the connective tissue and consistent acidophilic cytoplasm with a central nucleus. cisplatin inoculation induced massive degenerative changes, hypertrophy of muscle fibers, distortion in blood capillaries, disturbance in the trabeculae of the heart, and retrogressive lacerations in muscle fibers. Moreover, leukocyte infiltrations and vacuolated muscle fibers are visible. Remarkably, MMINA treatment protects heart tissue from cisplatin deteriorating alterations, with less capillary dilatation and vacuolar alterations in comparison to the cisplatin-alone group, and maximum muscle fibers appearance as control group signifying protecting action of MMINA usage.

The cerebellar cortex of control, DMSO, and MMINA alone treated groups indicated typical morphology with usual size and deeply basophilic Purkinje cells. Cisplatin inoculation provoked austere multifocal histological fluctuations in the cerebral cortex. The Purkinje cells of the cisplatin group were shrunken with some exhibiting features of karyolysis and had peri-cellular halos when compared with the control group. Many vacuoles of variable sizes either single or multiple appeared between and inside most of the cells in all layers. Co-treatments with MMINA significantly recused brain tissue damages induced by cisplatin in a dose-dependent response. Most of the pyramidal and granule cells were more or less like that of the control group. Very slight number of shrunken, darkly stained nuclei, pericellular halos, and the vacuolated neuropil were seen.

### Molecular interaction analysis

The computational bonding interaction analysis of COX-2, STAT3, and TNF-α were in cooperation with flow cytometry analysis of MMINA on the population of T cells. The inhibitor (2-(5-methoxy-2-methyl-1H-indol-3-yl)-N[(E)-(3-nitrophenyl) methylidene] acetohydrazide) (MMINA) was very well docked within the active site of COX-2, STAT3, and TNF-α.

**Figure 8:**
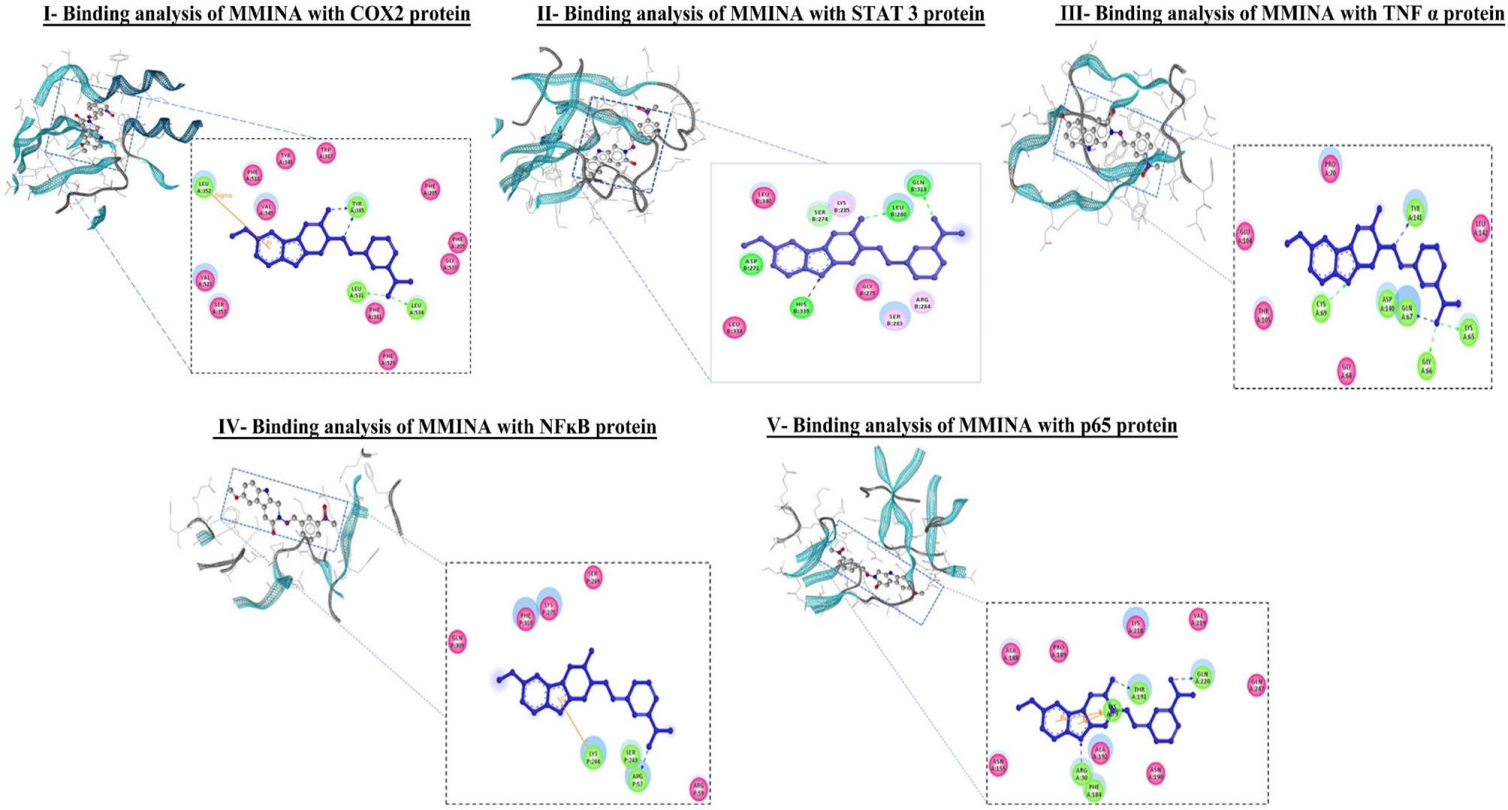
Binding analysis of MMINA with COX2, STAT3, TNF-α, NF-κB and p65protein. In the 3D confirmation, protein is shown in snake ribbon while the MMINA is shown in grey ball and stick model. In 2D representation blue ball and stick model represent the MMINA, residues involved in hydrophobic interactions are shown in pink disc while the residues involved in covalent and coordinate covalent interactions are shown in green discs. orange lines indicating pi-interactions in p65.

The docking interaction of MMINA with COX-2 revealed several molecular interactions as shown in Table 3 with the binding free energy of −9.00. LEU:531 and LEU:534 formed a covalent bond with anionic oxygen of NO_2_ of the benzene ring moiety while TYR:385 residue formed coordinate covalent bond with nitrogen and with the carbonyl oxygen moiety. LEU:352 residues of COX-2 protein were involved in pi-sigma interactions with the benzene ring i.e., –OCH_3_ (methoxy) moiety. Apart from these numbers of hydrophobic interactions were observed as shown in Table 3 (Fig. 8, I).

**Table 3:**
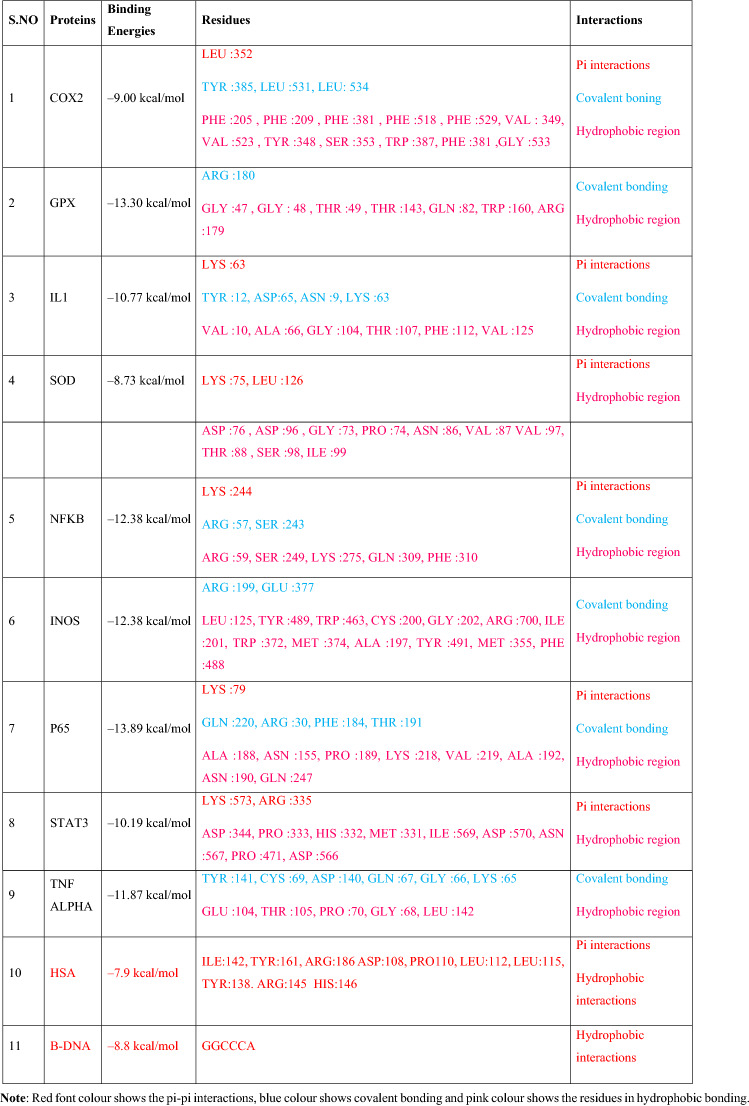
Binding residues and types of interactions of MMINA with all studied proteins and DNA.

Red font colour shows the pi-pi interactions, blue colour shows covalent bonding and pink colour shows the residues in hydrophobic bonding.

Binding analysis of MMINA with the STAT3 binding cavity revealed strong interactions with the binding free energy of −10.19. LEU:280 and GLN:318 were involved in hydrogen bonding with methoxy moiety of the MMINA compound. His:339 was involved in coordinate covalent interaction with acetohydrazide moiety of the lead compound. Apart from this Cyc:320, Gly:323, Leu:338, Leu:340, and Val:392 were involved in hydrophobic interactions with MMINA compound (Fig. 8, II) (Table 3).

The binding analysis of MMINA lead compound with TNF-α **s**howed strong binding (with the binding energy of −11.87 within the critical binding site of TNF-α a significant proinflammatory cytokine. TYR :141 residues of TNF-α was involved in covalent bonding with the nitrogen of methylidene moiety, CYS :69 was also involved in covalent bonding with acetohydrazide ring. While GLN :67, GLY :66 and LYS :65 were involved in covalent bonding with the nitrophenyl ring of MMINA. Critical amino acid (Table 3) of TNF-α was involved in hydrophobic interactions in keeping the drug within the active site (Fig. 8, III).

Binding of MMINA within the cavity of NF-κB revealed key residues of NF- κB e.g., LYS :244 ARG :57, SER :243 ARG :59, SER :249, LYS :275, GLN :309 and PHE :310 involved in covalent, coordinate covalent and Pi-Pi interactions with MMINA compound with the binding free energy of −10.34 kcal/mol (Fig. 8, IV) (Table 3). P65 the component of the NF-κB p65-c-Rel complex showed strong binding with MMINA compound with several strong molecular interactions and binding free energy of −13.89 kcal/mol (Table 4). GLN :220 of p65 formed H-bonding with a nitro group, while ARG :30 and PHE: 184 were made H-bonding with pyrrole ring of MMINA compound. Apart from these THR :191 also showed covalent interaction with carbonyl oxygen of amide group. LYS :79 was involved in pi-pi interactions with both pyrrole and benzene ring of MMINA compound. Hydrophobic interactions were observed involving number of binding site residues e.g. ALA :188, ASN :155, PRO :189, LYS :218, VAL :219, ALA :192, ASN :190, and GLN :247 (Fig. 8, V).

**Table 4:** Molecular descriptors of MMINA through in silico QSAR model.

Drug	Molecular weight	Num.heavy atoms	Fraction csp3	Num.rotatible bonds	Num. H bonds acceptor	Num. H bond donor	Molar refractivity	TPSA	Formula
Physiochemical properties	376.501 g/mol	59	0.16	10	3	3	104.33	87.450	C20H32N403
Pharmacokinetics	GI absorption	BBB permeant	P-gp substrate	CYP1A2 inhibitor	CYP2C19 inhibitor	CYP2C9 inhibitor	CYP2D6 inhibitor	CYP3A4 inhibitor	Log Kp (skin permeation)
	High	No	No	No	Yes	Yes	No	Yes	−6.33 cm/s
Water solubility	Log S (ESOL)	Solubility	Class	Log S (SILICOS-IT)	solubility	Class			
	−4.01	3.55e−02 mg/ml ; 9.69e−05 ml/l	Moderately soluble	−6.14	2.64e−04 mg/ml ; 7.19e−07 ml/l	Poorly soluble			
Druglikeness	Lipinski	Ghose	Veber	Egan	Muegge	Bioavailibility score			
	Yes, 0 violation	Yes	Yes	Yes	Yes	0.55			

**Figure 9:**
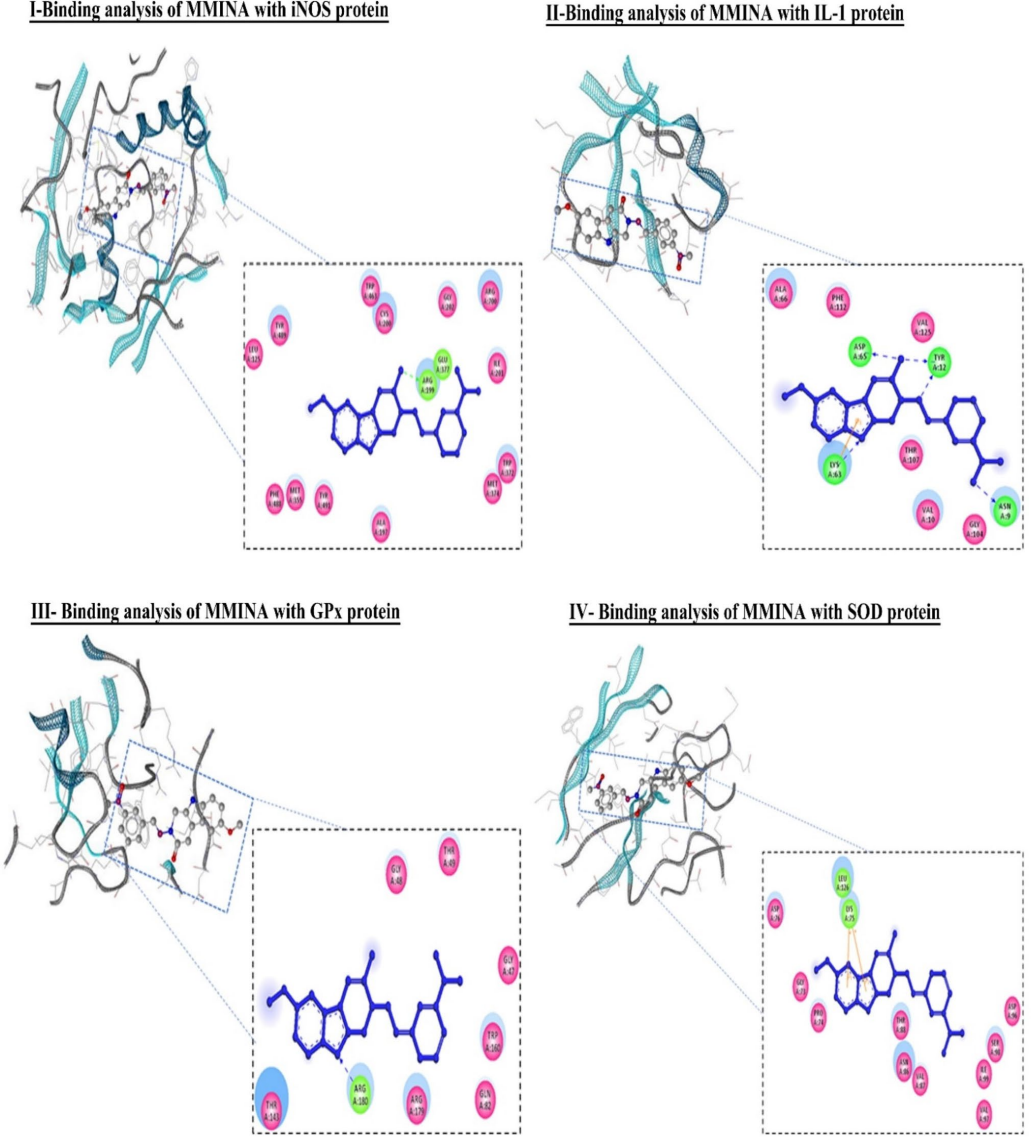
Binding analysis of MMINA with iNOS, IL-1, GPX and SOD protein. In the 3D confirmation, protein is shown in snake ribbon while the MMINA is shown in grey ball and stick model. Blue ball and stick model represent the MMINA in 2D conformation, residues involved in hydrophobic interactions are shown in pink disc (hydrophobic interactions in GPx, while the residues involved in covalent and coordinate covalent interactions are shown in green disc (covalent in GPx, H-bond in IL-1). Orange colour line in IL-1 was indicating pi interactions.

Molecular docking analysis revealed that MMINA drug was pretty well placed within the cavity of iNOS protein with binding energy −12.38 as shown in Table 3. ARG :199 and GLU :377 formed covalent bond acetohydrazine and nitrophenyl ring respectively. Similarly, number of key active site residues (LEU :125, TYR :489, TRP :463, CYS :200, GLY :202, ARG :700, ILE :201, TRP :372, MET :374, ALA :197, TYR :491, MET :355) were involved in hydrophobic interactions (Fig. 9, I).

Binding interaction analysis of MMINA compound with IL1 revealed strong binding interaction with 5 H-bond along with pi-pi stacking and hydrophobic interactions with a binding free energy of −10.77 kcal/mol as shown in Table 3. TYR :12 of IL formed two H-bond with the carbonyl oxygen of the amide and with the nitrogen of methylidene, similarly ASP :65 formed H-bond with amide moiety, ASN :9 was involved in bonding with oxygen of the nitro group while LYS :63 made both pi interactions and direct covalent bonding with the nitrogen of the indole moiety. VAL :10, ALA :66, GLY :104, THR :107, PHE :112 and VAL :125 residues were involved in hydrophobic interaction (Fig. 9, II).

Docking analysis of MMINA with GPx revealed strong binding with key interacting residues of GPx protein (GLY :47, GLY: 48, THR :49, THR :143, GLN :82, TRP :160, ARG :1769 and ARG :180) with binding energy of −13.30 kcal/mol (Table 3). ARG :180 of GPX formed H-bond with nitrogen of the indole moiety of MMINA. The drug molecule was kept very well in the binding site of GPx by several hydrophobic interacting residues e.g. GLY :47, GLY: 48, THR :49, THR :143, GLN :82, TRP :160 and ARG :179 (Fig. 9, III).

Molecular interaction analysis of MMINA with SOD1 indicated the strong binding with the binding free energy of the energy of −8.73 as shown in Table 3. LYS :75 and LEU :126 was involved in pi interactions with benzene and pyrrole ring of indole. ASP :76, ASP :96, GLY :73, PRO :74, ASN :86, VAL :87 VAL:97, THR:88, SER :98 and ILE :99 residues were involved in the hydrophobic region (Fig. 9, IV).

**Figure 10:**
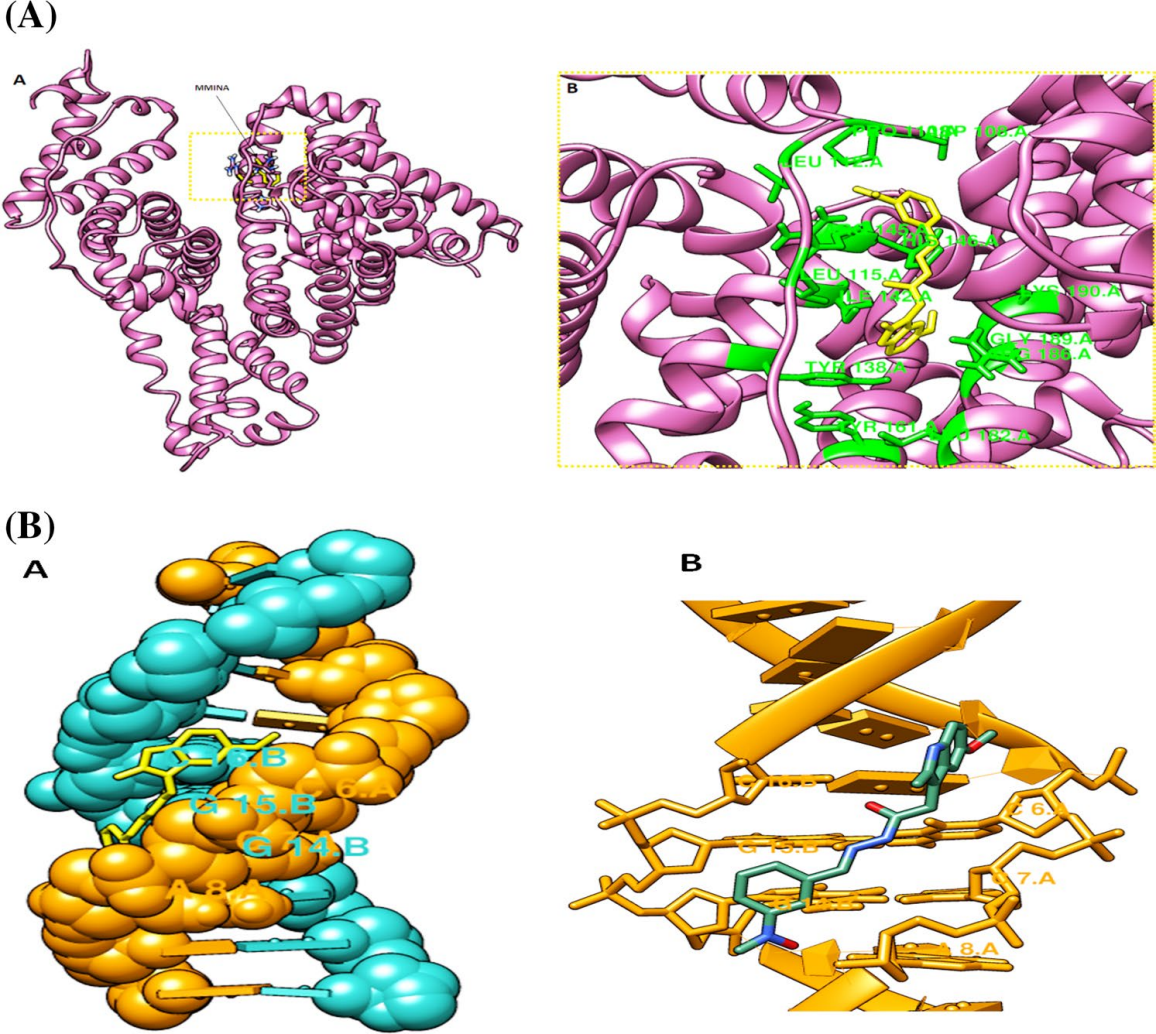
Molecular docking analysis of MMINA with human serum albumin (HSA). (**A**) HAS is represented in purple ribbon with bound MMINA shown in yellow stick model. Detail of molecular interactions between MMINA and HAS at residue level. Interacting residues are shown in green sticks while the black dotted lines represents hydrogen bonding. (**B**) Molecular docking analysis of MMINA with B-DNA. (**A**) DNA molecule is shown in hydrophobic representation with bound MMINA in yellow stick model. (**B**) Interacting di-deoxyribonucleotides are shown in yellow stick model and MMINA in green stick model.

Interaction analysis of MMINA with human serum albumin (HAS) revealved strong binding with the binding free energy of −7.9 kcal/mol. ILE:142, TYR:161 and ARG:186 made pi-pi and pi-sigma interactions respectively with important structural moieties od MMINA. ASP:108, PRO110, LEU:112, LEU:115, TYR:138. ARG:145 and HIS:146 residues of HSA made number of hydrophobic and vander waal interactions with MMINA compound (Fig. 10A).

Docking analysis of MMINA with the B-DNA revealved binding within the minor groove with binding free energy of −8.8 kcal/mol. Interaction analysis revealved binding of MMINA with GGC and CCA on side by side strand of DNA in minor groove (Fig. 10B).

#### QSAR model

**Figure 11:**
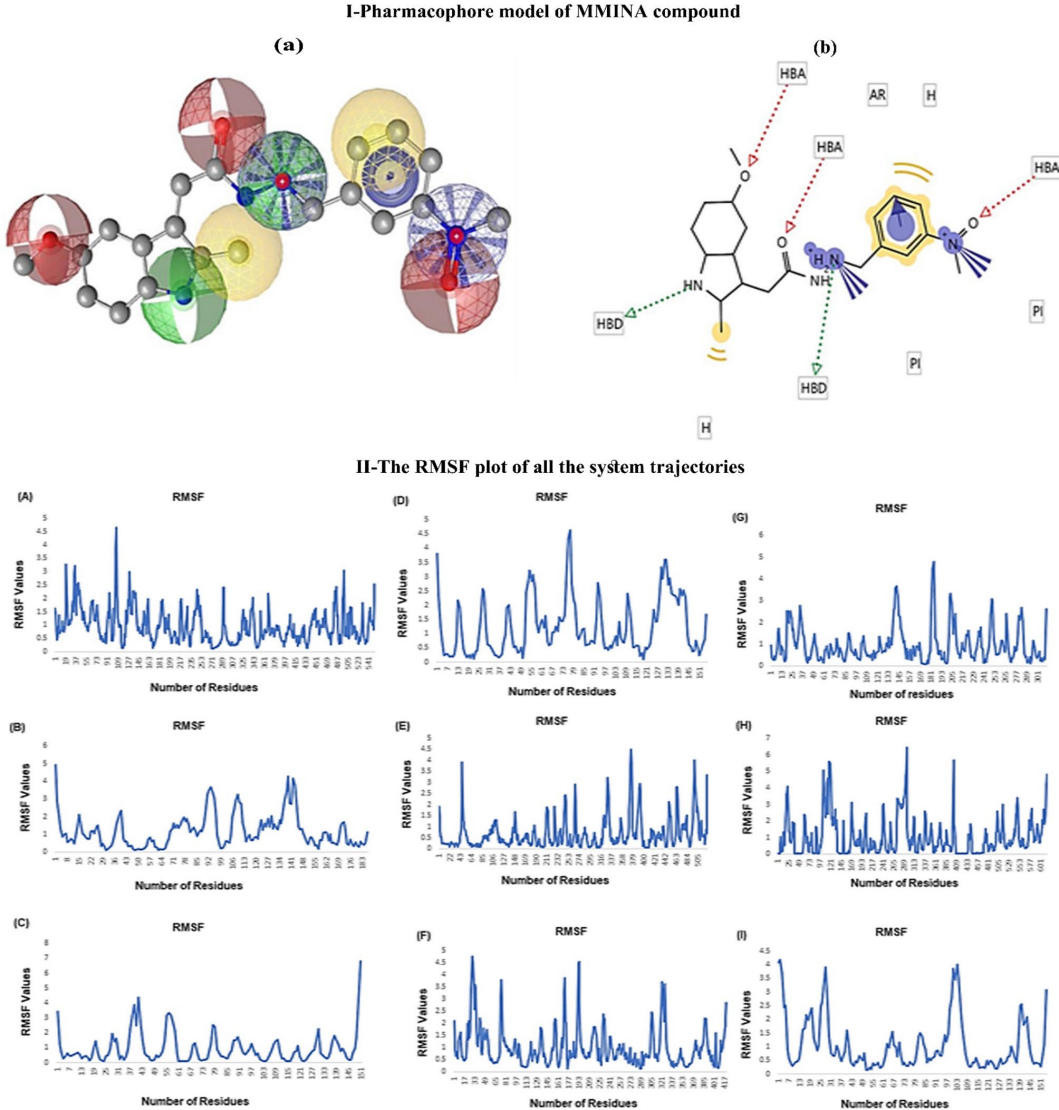
**I** Pharmacophore model of MMINA compound. (**A**) 3D representation of MMINA pharmacophore model. Red spheres repersents hydrogen bond acceptor (HBA), green spheres showing hydrogen bond donor (HBD) and yellow spheres depicting aromatic rings. (**B**) The same color theme with same descriptors are represented in 2D conformation. **II:** The RMSF plot of all the system trajectories. (**A**) COX2 (**B**) GPX, (**C**) IL1 (**D**) SOD, (**E**) STAT3, (**F**) iNOS, (**G**) NFKB, (**H**) p65 and (**I**) TNF-α.

To predict the physicochemical, biological, and environmental properties of MMINA compounds from the knowledge of its chemical structure QSAR (quantitative structure–activity relationship) model was computed (Fig. 11; I, Table 4). The molecular descriptors were calculated using computational tools. The molecular weight of the drug was 376.501 g/mol, the number of heavy atoms it contains was 59, the number of H bonds acceptor was 3, the number of H bond donor was 3 and its molecular formula was C20H32N4O3. The number of negative ionizable elements was 0 and positive ionizable elements were 2. The cLog *p* value was 3.292.

#### Molecular dynamic simulation

To measure the stability and structural behavior of all the docked systems molecular dynamics simulations were performed. The root means square fluctuation (RMSF) was estimated to measure the stability and fluctuations of protein C-alpha atoms of COX-2, IL1, SOD, GPX, NF-κB, P65, STAT3, iNOS, and TNF-α systems. An overall convergence of energies indicated well behaved-systems. The dynamics trajectories at 10 ns were generated to assess the stability and conformational switches of all the studied systems (Fig. 11; II). Detailed dynamic trajectories analysis revealed the overall stability of the COX-2 system in and around the binding site, the fluctuation was observed in the area encompassing residues 89–111 with RMSF value of 5. The dynamic trajectory of GPx system showed fluctuation at a different region of the protein i.e. 85–99, 106–113, and 134–141 with RMSF value 5 but the critical residues involved in binding with the compound showed stability. Detailed dynamic trajectory analysis of the IL1 system showed fluctuation between 1–7, 37–43, and 145–151 region of the protein. Interestingly all fluctuations were around the binding pocket to bring conformational changes to attain a favorable binding pose. The SOD protein showed fluctuation between 1–7, 73–79, and 127–133 residues but between 73–79 number of residues, the fluctuation was high with an RMSF value of 5. Analysis of the STAT3 system trajectory revealed fluctuations between 43–64, 358–379, and 484–505 region of the protein but the key interacting residues of the binding pocket were stable throughout the trajectory. The dynamic trajectory of iNOS protein showed fluctuation in the residues between17-33, 161–177, and 193–209. The dynamic trajectory of NF-κB revealed fluctuation in the region encompassing residues181-193 with an RMSF value of 5. The dynamic trajectory of P65 protein showed fluctuation in the region encompassing residues 97–121, 265–289, and 385–409 with an RMSF value of 6.5. TNF-α system trajectory showed fluctuation between 1–7, 25–31, and 97–103 number of residues with RMSF value 4. Conclusively, all the system trajectories showed stability in the binding region throughout the simulation experiments while region near and far from binding sites of all systems showed fluctuation to attain favorable binding conformation.

## Discussion

Explication of mechanisms involved in cisplatin chemotherapy side effects is essential to find out potential complementary compounds/drugs. The exact mechanism of cisplatin induce toxicity is still under investigation however, oxidative stress and inflammation are proposed to be significant causes in the pathophysiology of cisplatin-induced organ damage^32–34^. In the current investigation, we have assessed various enzymatic and molecular markers to delineate the protective mechanism of action of MMINA against cisplatin-persuaded tissue injury using a rodent model, furthermore, molecular docking studies were performed to study the drug targets. Our previous investigation revealed the anti-inflammatory, analgesic, and gastro-protective action of the novel compound (MMINA). The synthesized compound MMINA has shown significant anti-inflammatory and analgesic activity as compared to reference drug indomethacin. It was further tested for ulcerogenic index and was found to be having significant gastric sparing activity. The toxicity of the compound was found to be very low in toxicity screening. It was also found to be a potent inhibitor of COX-2 expression. Molecular docking of new indole derivative MMINA, a cyclooxygenase inhibitor to probe its binding mechanism with bovine serum albumin was also investigated. Cisplatin encouraged severe histopathological amendments in kidneys, like hemorrhage, congestion, hyaline casts formation, and coagulative necrosis^35^. These results support the previous examinations^36,37^. Serum urea and creatinine are biomarkers to assess the normal functioning capacity of renal tissue, the degree of assault, and the toxicity of a chemical compound on organs/tissues. Urea is the metabolic product of protein catabolism and a rise in serum urea might impede the kidney function if it is not controlled accordingly. The administration of cisplatin also increased the level of blood creatinine. It is well known that cisplatin accumulated in renal parenchymal cells increases the cisplatin drug load in the kidney than that in the blood^38^. The widespread renal damage results in the failure of creatinine clearance which results in the creatinine accumulation in the blood^39^. Our study confirms that MMINA prominently attenuates the cisplatin-induced reduction in kidney function and ameliorates the observed histological damage and that this protective effect is mediated noticeable attenuation of the cisplatin-induced proinflammatory response, oxidative stress and showed a marked reduction in the kidney function markers. While higher doses of cisplatin have unveiled higher effectiveness in cancer treatment nonetheless this likewise upsurges the risk of renal injuries^40^. Studies have illustrated that momentarily subsequently to the start of cisplatin treatment, signs of nephrotoxicity such as a reduction in glomerular filtration rate, and a rise in creatinine levels and blood urea nitrogen (BUN) are pragmatic^41^. Though not entirely understood, cisplatin-induced nephrotoxicity has been allied to a rise in ROS level and subsequently an intensification in Proteins, lipids, and nucleic acid oxidation and a reduction in glutathione peroxidase (GPx), and SOD, which are recognized as significant elements of antioxidant defense^42,43^.

Alanine aminotransferase (ALT), aspartate transaminase (AST), and alkaline phosphatase (ALP) are widely distributed throughout the body and found primarily in the heart, liver, skeletal muscle, and kidney. They are commonly used as indicators of liver function and sensitive markers of hepatocellular degenerative and necrotic changes^44^. Cisplatin hepatotoxicity was evidenced by elevated transaminases and other liver function markers along with histopathological manifestations, including degenerative changes, vacuolations, inflammatory cell infiltration, and others. Consistent with previous observations, we observed the elevation of serum transaminases in the cisplatin group along with histopathological manifestations^45,46^. Our study revealed that MMINA treatment ameliorated serum aminotransferases and maintained the normal architectural integrity of hepatocytes in rat models of cisplatin-induced injury. This was confirmed by decreased plasma ALT, AST, and ALP has taken together with the apparent typical liver histology. Also, MMINA treatment reduced the intensity of oxidative stress markers MDA and NO and increased the antioxidant markers GPx, and SOD in cisplatin-inoculated rats. Our finding seemed to be consistent with numerous earlier observations that demonstrated a hepatotoxic effect of cisplatin and its alliances with the amplified free radical formation and the consequent oxidative stress^45,46^. In the current experimental study model, our results illustrated that MMINA exerted an antioxidant effect on cisplatin-induced toxicity. The levels of tissue antioxidant parameters were significantly greater in the cisplatin + MMINA group when compared with the cisplatin group. In contrast, the levels of oxidative stress products (MDA, and NO) were considerably higher in the cisplatin group than the Cisplatin + MMINA group. Thus, our results proposed that MMINA diminishes cisplatin’s hepatotoxic effect by escalating antioxidant mechanisms.

Neurotoxicity is a recurrent confrontational effect of chemotherapies including cisplatin. Explanation of mechanisms concerned in cisplatin-induced neurotoxicity is significant to explorer the novel potential complementary therapy. Numerous mechanisms have been suggested nevertheless, inflammation and the oxidative injury appear to be the central mechanisms in the neurotoxicity induced by cisplatin^47,48^. Our study illustrated severe neurotoxicity induced by cisplatin in the rat brain which was demonstrated by the enhanced activities of AChE, changed antioxidative/ oxidative status, and inclined secretion of pro-inflammatory cytokines. TNF-α, NF-κB, IL-1β, and COX-2, are enormously effective and taken to be the foremost cytokines since they may likewise activate a cascade of events which in turn results in the proclamation of additional cytokines^47,49^. Enormous release of pro-inflammatory cytokines has been observed to be engaged in numerous neuropathological disorders^50^. Neuro-inflammation might be an additional likely mechanism in cisplatin-induced toxicity in the brain of rats. This was demonstrated in the current study by the increase in the levels of TNF-α, IL-1β, and iNOS in both the brain tissue as well as in the serum which supports previous findings^51^. NF-κB being a vital element that persuades the transcription of the pro-inflammatory genes and consequently plays a crucial part in the inflammation^52^. In our study, the cisplatin group alone have shown upregulation of NF-κB. hence may encouraging the overproduction of pro-inflammatory cytokines. Our results illustrated that treatment with MMINA decline the high levels of TNF-α, IL-1β, and COX-2 induced by cisplatin. This anti-inflammatory effect of MMINA by neutralizing NF-κB and the antioxidant activity shown by MMINA by enhancing GSH may be essential mechanisms behind the stated enhancement in the pro-inflammatory cytokines.

Numerous observations proposed that cisplatin-induced cardiotoxicity consequences because of the disposition of cisplatin in the sinoatrial node area which causes bradycardia, dysfunctioning of the left ventricle, and cardiomyocyte contraction^53^. These irregularities can be recognized as ER stress, oxidative stress, inflammation, and apoptosis. Our study illustrated that MMINA significantly, diminishes cisplatin-mediated cardiac damage, as noticed from the biochemical analysis of various cardiac markers like CK-MB, and LDH and severe histopathological amendments. MMINA decreases the cisplatin-induced ROS generation in the heart of rats exposed to cisplatin. MMINA ameliorates the cisplatin-induced inflammation through the suppression of proinflammatory cytokines (TNF-α, IL1), in the Cisplatin + MMINA group when compared with the cisplatin group.

Cisplatin treatment increases the expression of various proinflammatory biomarkers and apoptotic regulatory transcription factors in various tissues. We observed the ameliorating potential of MMINA at both gene and protein levels in the regulation of the expression of COX-2, TNF-α, IL1, STAT 3, NF-ĸB p65. Cyclooxygenase 2 (COX-2) is an enzyme mainly known for its activity in inflammation and pain and accountable for the assembly of prostaglandins, leading to a boost of inflammation through chemo-attraction to inflammatory cells and vasodilation^54,55^. Recently studies have revealed that the signal transducer and activator of transcription (STAT) 3 perform crucial for all recognized features of the IL-10-structured anti-inflammatory effect both in vivo and in vitro^56^. Being one of the utmost significant pro-inflammatory cytokines, TNF-α contributes to edema formation and vasodilatation, and leukocyte adhesion to epithelium via expression of adhesion molecules; it controls blood coagulation, subsidizes to oxidative stress in sites of inflammation, and indirectly induces fever^57^. Therefore, we evaluated the effect of MMINA on the configuration of the T-cell population by flow cytometry. NF-κB regulates the transcription of iNOS and COX-2 which are known for their activity as inflammatory mediators. Agents that constrain NF-κB leads to decreased expression to iNOS and consequently may have positive therapeutic results for the conduct of inflammatory diseases. Studies illustrated that indole derivatives are proficient in overwhelming NF-κB activation^58^. This supports our study which reveals that MMINA overwhelms COX-2 and iNOS probably through reticence of nuclear factor kappa B (NFκB) activation as observed in the immunoblot and densitometric analysis in the liver, brain, kidney, and heart tissues of rats. RT-PCR analysis also illustrated the decreased expression of COX-2, nuclear factor kappa B (NF-κB) and iNOS in the Cisplatin + MMINA group as compared to the control group. Also, our study suggested that MMINA inhibits NFκB activation and proinflammatory genes expression through overwhelming the phosphorylation of NF-κB p65 in liver, brain, kidney and heart tissues of cisplatin + MMINA group. Furthermore, our data revealed that cisplatin administration considerably inclined the level of expression of TNF-α, and IL1, as comprehended in the immunoblot and densitometric analysis 6 in the liver, brain, kidney, heart tissue of cisplatin + MMINA group as compared to cisplatin group. Also, RT-PCR analysis revealed an elevation in the expression of the TNF-α, and IL1. Administration of MMINA simultaneously with cisplatin considerably lowered the expression of TNF-α, and IL-1 in the liver, brain, kidney, heart tissue of cisplatin + MMINA group as compared to cisplatin group, reflecting the anti-inflammatory efficacy of MMINA. STAT3, is exceedingly interrelated with NF-κB signaling^11,59^. There are prominent counterparts, as well as dissimilarities, among STAT3 and NF-κB. Both proteins are not only insistently stimulated in cancer and crucial for transducing cytoplasmic signals from extracellular stimuli, but they are known for their activity as nuclear transcription factors essential for controlling genes that play an important part in angiogenesis. tumor proliferation, survival, and invasion, furthermore to genes encoding significant tumor-stimulating inflammatory mediators^59–61^. It is systematically significant that NF-κB and STAT3 correlate with each other at numerous stages in an exceedingly context-dependent fashion. Such as some inflammatory factors encoded by NF-κB target genes, like interleukin-6 (IL-6), are essential STAT3 activators^11,17, 59^. Eventually, STAT3 and NF-κB also co-regulate several oncogenic and inflammatory genes^17,62^. Studies also revealed that NF-κB activation plays a crucial role in IL-1β assembly, as most cytokines are allied with NF-κB^63^. Our study proposed that inhibition of NF-κB by MMINA treatment, the function of STAT3 is also inhibited, which in turn inhibits IL-1. In silico validation revealed that MMINA may protect against cisplatin-induced acute liver, brain, kidney, and heart damage. Molecular docking analysis predicted strong binding with the GPx, COX2, IL1, and NF-κB with stability in dynamic behaviors as well (Supplementary file S2; Fig. 3). The physiochemical properties of MMINA predicted it as a good drug-like molecule and its mechanism of action is predictably through inhibition of COX-2, STAT3, NF-κBp65, IL-1, TNF-α, and GPx.

## Conclusion

The key objective of the present study to contribute to the growing drug design area (such as treatment innovation and improvement) by examining protein-drug interactions. We aimed to present a summary of the protective effects of MMINA against cisplatin allied hepatotoxicity, neurotoxicity, cardiotoxicity, and nephrotoxicity, during cisplatin therapy. MMINA can compensate cisplatin-induced neurotoxicity, hepatotoxicity, neurotoxicity, cardiotoxicity, and nephrotoxicity frequently through anti-inflammatory and antioxidant and mechanisms. In this context, the protective effects of MMINA essentially root in a variation of STAT3, TNF-α, COX2, IL1, and NF-κB transcription factors which are significant regulators of inflammation and oxidative stress. Our comprehensive experimental and in silico studies provide novel insights into the protective activities of novel indole derivative MMINA and its potential uses in combination anticancer therapy. These promising possessions require proof-of-concept clinical trials to be conducted and additional efforts to test the protective effects of MMINA and its structural analogs.

## Methods

### Ethics statement

The study protocol was approved by the ethics committee in the Shaheed Benazir Bhutto Women University, Peshawar, Khyber Pakhtunkhwa, Pakistan. Studies reported in the manuscript were carried out in compliance with the ARRIVE guidelines.

### Animals

Adult male 35 Wister rats weighing 12–14 weeks old, 240–260 g, were obtained from the animal house of Department of the animal Sciences, Shaheed Benazir Bhutto Women University, Peshawar, Khyber Pakhtunkhwa, Pakistan. The rats were fed a dietary formulation of protein (18.1%), fat (7.1%), carbohydrate (59.3%), 125, and fiber (15.5%) with food and water being provided ad libitum. Animals were given favorable conditions (12/12 h light and dark cycle , temperature 25 °C, and humidity 60 ± 10% and pathogen-free environment)^64^.

### Biological characterization of MMINA

The methyl ester of indomethacin (0.01 mol) and hydrazine hydrate (99%) (0.2 mol) in presence of absolute ethanol (50 mL) were refluxed for 30 h. The reaction mixture was concentrated by using rota vapor and poured in a beaker containing crushed ice in small portions while stirring and kept for 4 h at room temperature. The solid was separated out by filtration. The product was dried and recrystallized from ethanol. The product was carefully checked by thin layer chromatography. The first compound was 2-(6-methoxy-2-methyl-1H-indol-3-yl) acetohydrazide compound **(1)**, and was obtained as the major product. The second compound, 4-chlorobenzohydrazide **(2)** was obtained as minor product. Both the compounds were fully characterized by the spectral data.

*2-(5-Methoxy-2-methyl-1H-indol-3-yl)-N'-[(E)-(3-nitrophenyl)methylidene] acetohydrazide* **(S3):** Yield: 68%; m.p.: 200‒202 °C; IR (KBr) cm^−1^: 3412 (NH), 3237 (C-H), 1654 (C = O), 1617 (C = N); ^1^H NMR (500 MHz, DMSO-d_6_): *δ* = 2.37 (3H, s, –CH_3_), 3.58 (2H, s, CH_2_), 3.74 (3H, s, –OCH_3_), 6.99–8.57 (7H, m, Ar–H), 10.63 (1H, s, = CH), 11.5 (1H, s, NH, D_2_O exchg.), 12.18 (1H, s, –CONH, D_2_O exchg.); ^13^C NMR (125 MHz, DMSO-d_6_): *δ* = 11.5 (CH_3_), 27.9 (CH_2_), 55.0, 100.2, 103.9, 109.4, 123.8, 124.2, 128.5, 130.3, 131.7, 133.3, 136.0, 140.33, 145.5, 148.1, 152.8, 162.2, 170.0; MS: *m/z* = 366.37 [M] + ; Analysis: for C_19_H_18_N_4_O_4_, calcd. C 62.29, H 4.95, N 15.29%; found C 62.36, H 4.93, N 15.24%^30^.

### Materials used

2-(5-Methoxy-2-methyl-1H-indole-3-yl)-N’-[(E)-(3-nitrophenyl)methylidene]acetohydrazide was synthesized by the department of pharmaceutical chemistry, College of Pharmacy, King Saud University 2457, Saudi Arabia^31,65,66^. The structure of the compound is shown in Fig. 1. Cisplatin was purchased from Sigma-Aldrich, USA. Antibodies against IL-1, STAT-3, TNF-α, NFĸB p65, COX-2, and β-actin were purchased from Thermo Fisher Scientific. Also, iNOS and SOD ELISA kits were purchased from My BioSource kits (Cat. # MBS 263,618 and MBS2548473) respectively.

### Experimental design and drug administration

The animals were divided into five groups and each group with seven rats.

Group1: Control (normal) group, received a single dose (i.p) of isotonic saline on the second day of the experiment.

Group 2: DMSO group, received a single dose (i.p) of 2% DMSO on the second day of the experiment.

Group 3: Cisplatin group, received a single dose of cisplatin (12 mg/kg i.p) on the second day of the experiment.

Group 4: Cisplatin + MMINA group (Cisplatin-MMINA), received MMINA (25 mg/kg i.p) dose for 7 days and a single dose of cisplatin (12 mg/kg) on the second day of the experiment, 1 h after the dose of MMINA.

Group 5: MMINA group, received MMINA (25 mg/kg i.p) dose for 7 days.

The doses of MMINA and cisplatin were selected after performing the pilot experiment.

### Sample collection and preparation

On the last day of the experiment, all animals were euthanized by injecting ketamine/xylazine mixture (75/2.5 mg/kg, respectively) via the intraperitoneal route^67^. Anesthetized rats were secured in a supine position and the blood was withdrawn via cardiac puncture and organ samples were taken from the liver, kidney, heart, and brain. Blood samples were collected for different biochemical measurements. A small portion of blood was used for flow cytometer analysis while the remaining was centrifuged for 15 min at 1200×*g* at 40 °C to separate serum and were stored at −70 °C. The brain, kidney, heart, and liver tissues were quickly harvested. One part of the tissues were fixed in 10% formalin for histopathological studies and the other part treated with liquid nitrogen was used for RNA extraction and Immunoblotting.

### Biochemical analysis of serum

The serum was used for the estimation of aspartate aminotransferase (AST), Alanine aminotransferase (ALT), Alkaline phosphatase (ALP), Creatine, Creatine kinase-MB (CK-MB), low-density lipoproteins (LDL), Alkaline phosphatase (ALP), and Urea by AMP diagnostic kits according to the instruction of the manufactures.

### Antioxidant status and oxidative stress markers

NO activity was measured by using My BioSource kits (Cat. # MBS480450). Afsar et al. methods were used to assess SOD and GPx activity^45,46^.

### Malondialdehyde (MDA) measurement

In the brain, heart, kidney, and liver tissue, the MDA levels were resolute as a sign of lipid peroxidation as described previously The Brain, heart, kidney, and liver tissues were homogenized in KCL solution (1.15% w/v). A reaction mixture 200ul SDS (8.1% w/v), 1.5 mL of acetic acid (20% v/v pH 3.5), 1.5 mL of thiobarbituric acid (0.8% w/v), and distilled water (700 µL). Samples were boiled for 1 h at 95 °C using glass balls as condensers. Samples were cooled under tap water and then centrifuged for 10 min at 4000 g. The absorbance of the supernatant was recorded at 650 nm using a spectrophotometer.

### Evaluation of acetylcholinesterase (AChE) activity in the brain

The brain acetylcholinesterase activity was tested by the procedure described by Ellman et al.^68^. Briefly, the test solution contained distilled water (135 μL), 100 mM potassium phosphate buffer (pH 7.4, 20 μL), 10 mM DTNB (20 μL), diluted sample (1:10 v/v, 5 μL), and 8 mM acetylthiocholine (20 μL) as a substrate. The deterioration of acetylthiocholine iodide activity was examined for 5 min (30 s intervals) at 412 nm and the results were presented as μmol/min/mg protein.

### Flow Cytometry Analysis of TNF α, COX-2, and STAT3 expression in Whole Blood Cells.

For flow cytometry analysis of TNF-α, COX-2, and STAT3 expression, whole blood was withdrawn via cardiac puncture. Aimed against CD4, monoclonal antibody conjugated to a fluorochrome was added to 100 μL of whole blood and then by whole blood lysing reagent (BD Biosciences, USA), the mixture was lysed. The supernatant was aspirated after centrifugation for 5 min at 300 g. Then to the pellet, 1 × fixation /permeabilization solution (500 µL, BD Biosciences, USA) was added and incubated in the dark at room temperature for 10 min. The cells were centrifuged for 5 min at 300 g and the supernatant was aspirated. Then to the pellet, 1 × fixation /permeabilization solution (500 µL) and FcR blocking reagents (10 µl, Miltenyi Biotech) was added following incubation at room temperature in the dark for 10 min after washing with 3 mL of wash buffer, cytokine-specific antibodies (20 μL) against TNF α, (Cat. # MP6-XT22), COX-2 (Cat. # PA1-9032), and STAT3 (Cat. # PY705) (Thermo fisher and BD Biosciences, USA) were added to the cells and following incubation at room temperature in the dark for 30 min. The expression of phenotypic markers of all cells was analyzed on a Beckman Coulter flow cytometer (Beckman Coulter, USA) using Cytomics FC 500 software 56. To analyze the staining of cell surface markers, the lymphocytes were first gated by their physical properties (forward and side scatter), then a second gate was drawn based on immunofluorescence characteristics of the gated cells.

### Gene expression analysis

Total RNA from the frozen liver, kidney, heart, and Brain tissue of each rat was extracted by using a kit, according to the manufacturer's instructions (Promega Cat. # Z3101). The cDNA synthesis was performed using the Abi kit. The reaction mixture was prepared to contain 10 µl fastStart Universal SYBR Green Master (Roche, Germany), 6 µM reverse primers, and 10 µg cDNA, with RNAase free water added to a total volume of 20 µl. The amplification and real-time analysis were done for 40 cycles with the following factors; 95 °C (10 min) to activate of FastStart Taq DNA polymerase; 60 °C (1 min) for amplification and real-time analysis^69^. The gene expression levels were determined using 2^-ΔΔCT^. Primer sequences used are shown in Supplimentary file S1;Table 1.

### Extraction of protein and Western blot analysis

Briefly, rat liver, kidney, brain, and heart tissue samples were lysed in RIPA buffer supplemented with freshly added protease and phosphatase inhibitor cocktail 1:100 (Sigma), and protein concentration was determined by Bradford assay^70^. The previously developed procedures with slight modifications were used to perform SDS-PAGE and western blot investigations^71,72^. For SDS-Page, equal amounts of proteins were loaded using BIO-RAD Mini protein TGX precast gels on the bio-rad Mini protein tetra system (10,025,025 REVA). After protein separation by gel electrophoresis, the proteins were transferred to PVD membranes using bio-rad trans blot Turbo transfer system. PVD membranes (bio-rad) were blocked for 2 h with either 5% BSA (Sigma) or 5% nonfat dry milk (Bio-Rad, Cat. #170–6404) then incubated with primary antibody overnight at 4 °C. The following antibodies were used: NFκB p65 (ThermoFisher Scientific Catalog # 2A12A7), STAT3 (Thermofisher Scientific Cat. # MA1-13,042), COX-2 (Invitrogen Cat. # PA1-9032), IL-1 (Abcam Cat. # ab2105), TNF-α (Abcam Cat. # EPR19147) and β-actin (Abcam Cat. # ab49900). The antibodies signals were detected by ECL western blotting substrate (bio-rad) and blots were visualized using the bio-rad Gel documentation system (ChemiDoc MP System).

### Histopathologic evaluation

Tissue samples of liver, heart, kidney, and brain were fixed in 10% formalin for 24 h and paraffin blocks were processed for microscopic examination. 3–5 µm slices were obtained from the prepared blocks and hematoxylin–eosin (H&E) was used for staining. Slides were visualized using a microscope (Nikon eclipse Ni Tokyo, Japan) at a magnification of 40 X.

### Molecular Structure of ligand and receptors

The three-dimensional structure of iNOS (PDBID: 1NSI), NFκB (PDBID: 1SVC), TNF-α (PDBID: 5yoy), p65 (PDBID:1NFI), GPX (PDBID: 2F8A), SOD (PDBID:5YTO), IL1(PDBID:5UC6), STAT3 (PDBID: 6NJS), COX-2 (PDBID:5F19), human serum albumin (HSA) (PDBID:5UJB) and B-DNA (PDBID:334D) were retrieved from protein data bank (PDB) (https://www.rcsb.org). Structure of ligand (2-(5-methoxy-2-methyl-1H-indol-3-yl)-N'-[(E)-(3-nitrophenyl) methylidene] acetohydrazide) was generated from the ChemDraw (www.cambridgesoft.com). Energy minimization and structure refinements were made using GROMMACS available in Chimera 1.5.6^73^, and VEGA ZZ (http://www.ddl.unimi.it).

### Molecular docking

Molecular docking of MMINA, (2-(5-methoxy-2-methyl-1H-indol-3-yl)-N'-[(E)-(3-nitrophenyl) methylidene] acetohydrazide) compound with iNOS, NFκB, TNF α, p65, GPX, SOD, IL1, COX-2, STAT3, HSA and B-DNA were carried out using AutoDock4.0^74^. The polar hydrogen atom and kollamen charges were added. Annealing parameters for hydrogen bonding and Van der Waals interactions were set to 4.0 A° and 2.5 A°. The number of runs for each docking experiment was set to 100 with the default remaining docking parameters. After docking best-docked complexes were carefully chosen based on binding free energy value and using Discovery Studio visualizer (http://accelrys.com/products/collaborative-science/biovia-discovery-studio) and UCSF Chimera^73^ molecular interactions were assessed.

### Molecular dynamic simulations

Molecular dynamics (MD) simulations experiments were performed to evaluate the stability and structural behavior of individual best-docked complex for each target molecule. To assess the stability and fluctuations of protein C-alpha atoms, time series of root mean square deviation (RMSD) and root mean square fluctuation (RMSF) were evaluated using the GROMACS 4.5 package^75^. All systems were solvated by the TIP4P water model^76^ followed by the addition of Na^+^ and Cl^ˉ^ counter ions to neutralize all the systems. To the end of the simulations experiments VMD^77^, PyMol (http://www.pymol.org), and GROMACS tools were used to investigate the stability and fluctuation behavior of all the systems.

### Pharmacokinetics study

Molecular descriptors and drug likeliness properties of the MMINA compound were analyzed using the Molinspiration server (http://www.molinspiration.com), based on the Lipinski Rules of five^78^. AdmetSAR (http://www.admetexp.org) database was used to assess the Absorption, Distribution, Metabolism, Excretion, and the toxicity of the compound. To find any objectionable toxic properties of the MMINA compound, Osiris Property Explorer (http://www.organicchemistry.org/prog/peo/) was used. Pharmacophore features were also measured using Ligand Scout 3.0b (www.inteligand.com).

### Ethical approval and consent to participate

The study protocol was approved by the ethics committee in the Shaheed Benazir Bhutto Women University, Peshawar, Khyber Pakhtunkhwa, Pakistan. Studies reported in the manuscript fully meet the criteria for animal studies specified in the ACS ethical Guidelines.

## Supplementary information

Supplementary information.

## Data Availability

All the data is contained in the manuscript.

## Abbreviations

MMINA: 2-(5-Methoxy-2-methyl-1H-indol-3-yl)-N'-[(E)-(3-nitrophenyl) methylidene] acetohydrazide
MDA: Malondialdehyde
GPx: Glutathione peroxidase
SOD: Superoxide dismutase
STAT3: Activator of transcription
IL-1: Interleukin- 1
NF-κB: Nuclear factor-κB
COX-2: Cyclooxygenase enzyme
iNOS: Inducible nitric oxide synthase
ROS: Reactive oxygen species
TLR-4: Toll-like receptor-4
LPO: Lipid peroxidation
NSAID: Non-steroid anti-inflammatory drugs
QSAR: Quantitative structure–activity relationship
ALT: Alanine aminotransferase
AST: Aspartate aminotransferase
ALP: Alkaline phosphatase
LFTs: Liver function tests
Cr: Creatinine
TGAs: Triglycerides
LDL: Low-density lipoprotein
HDL: High-density lipoprotein
