# Supplementary material for: Molecular docking, pharmacokinetic studies, and in vivo pharmacological study of indole derivative 2-(5-methoxy-2-methyl-1H-indole-3-yl)-N′-[(E)-(3-nitrophenyl) methylidene] acetohydrazide as a promising chemoprotective agent against cisplatin induced organ damage
Source: Sci Rep. 2021 Mar 18;11:6245. doi: 10.1038/s41598-021-84748-y (PMC7973782; doi:10.1038/s41598-021-84748-y)
Supplement: Supplementary file 1 — Supplementary information. [file 41598_2021_84748_MOESM1_ESM.pdf]

Supplementary file S1:

Molecular docking, pharmacokinetic studies, and in vivo pharmacological study of indole derivative 2-(5-Methoxy-2-methyl-1H-indole-3-yl)-N'-[(E)-(3-nitrophenyl) methylidene] acetohydrazide as a promising chemoprotective agent against Cisplatin induced organ damage

Suhail Razak[1*, #], Tayyaba Afsar[1#], Nousheen Bibi[2#], Mahmoud Abulmeaty[1], Wajhul Qamar[3], Ali Almajwal[1], Anam Inam[2], Dara Al Disi[1,] Mashooq Ahmad Bhat[4]

Supplementary Table 1: Primers

Candidate gene	primer
**NF-κB p65**	F: 5′- ACACCTCTGCATATAGCGGC-3′<br>R: 5′- GGTACCCCCAGAGACCTCAT-3′
**TNF-α**	F: 5′-GCGGAGTCCGGGCAGGTCTA-3′<br>R: 5′-GGGGGCTGGCTCTGTGAGGA-3′
**COX-2**	F: 5′-CACTCATGAGCAGTCCCCTC-3′<br>R: 5′-ACCCTGGTCGGTTTGATGTT-3′
**IL-1**	F: 5′-ACCTGCTCCACTGCCTTGCT-3′<br>R: 5′-GGTTGCCAAGCCTTATCGGA-3/
**STAT-3**	F: 5′-CCCCCGTACCTGAAGACCAAG-3′<br>R: 5′-TCCTCACATGGGGGAGGTAG-3′
**iNOs**	F: 5′-CTATGGCCGCTTTGATGTGC-3′<br>R: 5′-CAACCTTGGTGTTGAAGGCG-3′

Supplementary Table 2: Effect of MMINA treatment on Total cholesterol, HDL, LDL and TGAs

Treatments	Total cholesterol (mg/dl)	HDL (mg/dl)	LDL (mg/dl)	TGAs (mg/dl)
**Control**	72.60 ± 1.71	35.50 ± 0.63	46.35 ± 0.86	71.08 ± 1.01
**DMSO Vehicle**	73.79 ± 1.73	35.59 ± 1.52	46.50 ± 1.03	71.79 ± 1.98
**Cisplatin (12.5 mg/kg)**	120.50 ± 2.82****	11.18 ± 0.83****	117.17 ± 2.45****	101.89 ± 2.88****
**Cisplatin + MMINA**	76.54 ± 2.01^{++++}	30.17 ± 0.67^{++++}	50.31 ± 0.70^{++++}	79.99 ± 1.24*, $^{++++}$
**MMINA(25 mg/kg)**	70.37 ± 2.44^{++++}	36.01 ± 0.91^{++++}	42.76 ± 0.81^{++++}	68.45 ± 1.14^{++++}

Data are mean ± SEM, (n = 7). *, **** p<0.05 and P < 0.0001 versus Control respectively, and $^{++++}$P < 0.0001 versus CP. Data analyzed by One-way ANOVA followed by Tukey’s multiple comparison tests.

Supplementary Table 3

Report generated by pathologist

Dr. Abdul Malik Al Sheikh (Pathologist), MD, FRCPC

Supplementary Table 3: Slide review: Preliminary observations

**Organ**	Control	MMINA treated
**Liver**	1. Minimal randomly scattered mononuclear and supportive hepatitis<br>2. Minimal lymphoplasmacytic and histolytic portal hepatitis.<br>3. Sinusoidal brown pigment accumulation interpreted as probable artifact of red blood cell staining.	1. Capsular fibrosis and mild chronic mononuclear and mildly suppurative inflammation (suggestive of peritonitis).<br>2. Minimal mononuclear portal hepatitis.<br>3. Mildly enhanced hepatocellular mitotic rate, presumptive.<br>4. Locally extensive moderate accumulation of pigment laden macrophages/Kupffer cells<br>6. Minimal extra-medullary hematopoiesis
**Brain**	Extensive dark neuron artifact interpreted as an artifact of dissection. There are extremely rare mild extravasations of blood into Virchow-Robbins' space. The habenular nuclei have a mesh-work of cells (presumptive neurons) with smudged nuclear features.<br>Diagnoses:<br>1. Locally extensive nuclear smudging in the habenular nuclei (a finding of uncertain significance).<br>2. Minimal extravasations of blood into Virchow-Robbins' space.	There is fairly extensive dark neuron artifact (presumptive secondary to dissection).
**Heart**	There are rare individual cardiac myocytes with increased cytoplasmic eosinophilia and bland darkly staining contracted nuclei.<br>Diagnoses:<br>1. Minimal individual myocytes change, interpreted as probable degerative change.	There are rare individual cardiac myocytes with slightly more darkly eosinophilic cytoplasm than neighboring cells and with more homogenous and darkly eosinophilic chromatin staining in contracted and shrunken nuclei.<br>Diagnoses:<br>1. Minimal individual myocytes change, interpreted as probable degerative change.

**Kidney**	No significant histological lesions are noted.	The renal capsule is segmentally broadened with fibrous connective tissue that is occasionally infiltrated with small numbers of mononuclear leukocytes and neutrophils.<br>Diagnoses:<br>1. Capsular fibrosis and mild chronic mononuclear and mildly suppurative inflammation (suggestive of peritonitis)

Supplementary File: S2

Molecular docking, pharmacokinetic studies, and in vivo pharmacological study of indole derivative 2-(5-Methoxy-2-methyl-1H-indole-3-yl)-N'-[(E)-(3-nitrophenyl) methylidene] acetohydrazide as a promising chemoprotective agent against Cisplatin induced organ damage

Suhail Razak[1*, #], Tayyaba Afsar[1#], Nousheen Bibi[2#], Mahmoud Abulmeaty[1], Wajhul Qamar[3], Ali Almajwal[1], Anam Inam[2], Mashooq Ahmad Bhat[4], Dara Al Disi[1],

Supplementary: Biological Characterization of MMINA

Synthesis of 2-(6-Methoxy-2-methyl-1H-indol-3-yl) acetohydrazide **(1)**

The methyl ester of indomethacin (0.01 mol) and hydrazine hydrate (99%) (0.2 mol) in presence of absolute ethanol (50 mL) were refluxed for 30 hours. The reaction mixture was concentrated by using rota vapor and poured in a beaker containing crushed ice in small portions while stirring and kept for 4 hours at room temperature. The solid was separated out by filtration. The product was dried and recrystallized from ethanol. The product was carefully checked by thin layer chromatography. The first compound was 2-(6-methoxy-2-methyl-1H-indol-3-yl) acetohydrazide compound **(1)**, and was obtained as the major product. The second compound, 4-chlorobenzohydrazide **(2)** was obtained as minor product. Both the compounds were fully characterized by the spectral data.

2-(6-Methoxy-2-methyl-1*H*-indol-3-yl) acetohydrazide (**1**). Color: white; Yield: 70%; m.p.: 168–170 °C; UV λmax (Methanol) = 280 nm; ^{1}H NMR (500 MHz, DMSO-d$_6$): δ = 2.38 (3H, s, CH_3), 3.54 (2H, s, CH_2), 3.80 (3H, s, OCH_3), 4.26 (2H, s, NH_2, D_2O exchg.), 6.67 (1H, d, J = 8.5 Hz, Ar-H), 7.16 (2H, d, J = 7.5 Hz, Ar-H), 9.16 (1H, s, NH, D_2O exchg.), 10.62 (1H, s, CONH, D_2O exchg.); ^{13}C NMR (125 MHz, DMSO-d$_6$): δ = 12.0 (CH_3), 30.2 (CH_2), 55.8 (OCH_3), 101.1, 105.1, 109.8, 110.0, 111.7, 128.0, 129.3, 129.7, 130.6, 134.3, 153.4, 170.8 (C=O); MS: m/z = 233.11 $[M]^+$, 234.07 $[M+1]^+$; Analysis: $C_{12}H_{15}N_3O_2$ for, calcd. C 61.79, H 6.48, N 18.01 %; found C 61.58, H 6.46, N 18.05 %.

4-Chlorobenzohydrazide **(2)**. Color: white; Yield: 20%; M.p.: 148–150 °C; UV λmax (Methanol) = 230 nm; ^{1}H NMR (500 MHz, DMSO-d$_6$): δ = 4.53 (2H, s, NH_2, D_2O exchg.), 7.52 (2H, d, J = 8.5 Hz, Ar-H), 7.84 (2H, d, J = 8.5 Hz, Ar-H), 9.87 (1H, s, CONH, D_2O exchg.); ^{13}C NMR (125 MHz, DMSO-d$_6$): δ = 128.86, 129.32, 132.50, 136.25, 165.29; MS: m/z = 170.45 $[M]^+$; Analysis: $C_7H_7N_2OCl$ for, calcd. C 49.28, H 4.14, N 16.42%; found C 49.37, H 4.12, N 16.46%.

3.3. *General procedure for the synthesis of 2-(5-methoxy-2-methyl-1-indol-3yl)-N-[(E)-substituted phenyl methylidine] aceto hydrazide derivatives* **(S1-S18)**. A solution of indole hydrazide **(1)** (371 mg, 1.0 mmol) in EtOH (15 mL) containing an appropriate substituted benzaldehyde (1.1 mmol) and a catalytic amount of Glacial acetic acid was heated under reflux for 3 hours. After cooling, 5 mL of water was added to the mixture and kept in a refrigerator for 12 hours. The product was obtained by filtration. The compound was washed several times with cold water. The compound obtained was recrystallized from ethanol.

2-(5-Methoxy-2-methyl-1H-indol-3-yl)-N'-[(E)-phenylmethylidene] acetohydrazide **(S1):** Yield: 70%; m.p.: 170–172 °C; IR (KBr) cm^{-1}: 3412 (NH), 3024 (C-H), 1654 (C=O), 1637 (C=N); ^{1}H NMR (500 MHz, DMSO-d$_6$): δ = 2.37 (3H, s, -CH_3), 3.55 (2H, s, CH_2), 3.74 (3H, s, -OCH_3), 6.65-8.00 (8H, m, Ar-H), 10.62 (1H, s, =CH), 11.26 (1H, s, -NH, D_2O exchg.), 11.9 (1H, s, CONH, D_2O exchg.); ^{13}C NMR (125 MHz, DMSO-d$_6$): δ = 12.2 (CH_3), 28.2 (CH_2), 55.5 (OCH_3), 100.7, 104.7, 110.0, 111.3, 127.2, 127.4, 127.6, 129.0, 129.2, 129.3, 130.0, 134.3, 134.8,

153.3, 167.7 (C=O); MS: *m/z* = 321.37 $[M]^+$; Analysis: for $C_{19}H_{19}N_3O_2$, calcd. C 71.01, H 5.96, N 13.08 %; found C 71.25, H 5.94, N 13.11%.

2-(5-Methoxy-2-methyl-1H-indol-3-yl)-N′-[(E)-(4-nitrophenyl)methylidene] acetohydrazide **(S2):** Yield: 75%; m.p.: 220–222 °C; IR (KBr) cm^{-1}: 3411 (NH), 3000 (C-H), 1654 (C=O), 1617 (C=N); ^{1}H NMR (500 MHz, DMSO-d_6): δ = 2.37 (3H, s, $-CH_3$), 3.58 (2H, s, CH_2), 3.74 (3H, s, $-OCH_3$), 6.97-8.26 (7H, m, Ar-H), 10.63 (1H, s, =CH), 11.70 (1H, s, -NH, D_2O exchg.), 12.2 (1H, s, -CONH, D_2O exchg.); ^{13}C NMR (125 MHz, DMSO-d_6): δ = 11.6 (CH_3), 28.2 (CH_2), 55.0 (OCH_3), 100.2, 110.8, 123.9, 124.0, 127.6, 128.0, 128.6, 129.6, 140.4, 142.0, 143.0, 144.0, 145.5, 151.0, 163.0, 175.0; MS: *m/z* = 366.37 $[M]^+$; Analysis: for $C_{19}H_{18}N_4O_4$, calcd. C 62.29, H 4.95, N 15.29 %; found C 62.14, H 4.97, N 15.25 %.

2-(5-Methoxy-2-methyl-1H-indol-3-yl)-N′-[(E)-(3-nitrophenyl)methylidene] acetohydrazide **(S3):** Yield: 68%; m.p.: 200–202°C; IR (KBr) cm^{-1}: 3412 (NH), 3237 (C-H), 1654 (C=O), 1617 (C=N); ^{1}H NMR (500 MHz, DMSO-d_6): δ = 2.37 (3H, s, $-CH_3$), 3.58 (2H, s, CH_2), 3.74 (3H, s, $-OCH_3$), 6.99-8.57 (7H, m, Ar-H), 10.63 (1H, s, =CH), 11.5 (1H, s, NH, D_2O exchg.), 12.18 (1H, s, -CONH, D_2O exchg.); ^{13}C NMR (125 MHz, DMSO-d_6): δ = 11.5 (CH_3), 27.9 (CH_2), 55.0, 100.2, 103.9, 109.4, 123.8, 124.2, 128.5, 130.3, 131.7, 133.3, 136.0, 140.33, 145.5, 148.1, 152.8, 162.2, 170.0; MS: *m/z* = 366.37 [M]+; Analysis: for $C_{19}H_{18}N_4O_4$, calcd. C 62.29, H 4.95, N 15.29 %; found C 62.36, H 4.93, N 15.24 %.

2-(5-Methoxy-2-methyl-1H-indol-3-yl)-N′-[(E)-(2-nitrophenyl)methylidene] acetohydrazide **(S4):** Yield: 70%; m.p.: 210–212°C; IR (KBr) cm^{-1}: 3407 (NH), 3063 (C-H), 1654 (C=O), 1617 (C=N); ^{1}H NMR (500 MHz, DMSO-d_6): δ = 2.37 (3H, s, $-CH_3$), 3.58 (2H, s, CH_2), 3.74 (3H, s, $-OCH_3$), 6.98-8.25 (7H, m, Ar-H), 10.63 (1H, s, =CH), 11.90 (1H, s, -NH, D_2O exchg.), 12.10 (1H, s, -CONH, D2O exchg.); ^{13}C NMR (125 MHz, DMSO-d_6): δ = 11.60 (CH_3), 27.8 (CH_2), 54.9 (OCH_3), 100.0, 103.8, 109.4, 110.6, 124.5, 127.8, 129.6, 133.4, 134.0, 141.4, 143.2, 147.9, 148.2, 152.0; 167.0, 170.0; MS: *m/z* = 366.37 [M]+; Analysis: for $C_{19}H_{18}N_4O_4$, calcd. C 62.29, H 4.95, N 15.29 %; found C 62.15, H 4.97, N 15.24 %

N′-[(E)-(4-chlorophenyl)methylidene]-2-(5-methoxy-2-methyl-1H-indol-3-yl)acetohydrazide **(S5):** Yield: 80%; m.p.: 180–182 °C; IR (KBr) cm^{-1}: 3411 (NH), 3071 (C-H), 1654 (C=O), 1597 (C=N); ^{1}H NMR (500 MHz, DMSO-d_6): δ = 2.36 (3H, s, $-CH_3$), 3.55 (2H, s, CH_2), 3.74 (3H, s, $-OCH_3$), 6.60-8.45 (7H, m, Ar-H), 10.63 (1H, s, =CH), 11.3 (1H, s, NH, D_2O exchg.), 12.00 (1H, s, -CONH, D_2O exchg.); ^{13}C NMR (125 MHz, DMSO-d_6): δ = 11.6 (CH_3), 28.2 (CH_2), 55.1 (OCH_3), 101.0, 128.5, 128.7, 128.8, 128.9, 129.5, 136.6, 146.7, 148.0, 149.0, 150.0, 151.0, 152.1,162.7, 172.0; MS: *m/z* = 355.81 $[M]^+$; Analysis: for $C_{19}H_{18}N_3O_2Cl$, calcd. C 64.13, H 5.10, N 11.81 %; found C 64.33, H 5.12, N 11.83 %.

N′-[(E)-(2,4-dichlorophenyl)methylidene]-2-(5-methoxy-2-methyl-1H-indol-3-yl)acetohydrazide **(S6)**: Yield: 65%; m.p.: 238–240 °C; IR (KBr) cm^{-1}: 3411 (NH), 2940 (C-H), 1654 (C=O), 1617 (C=N); ^{1}H NMR (500 MHz, DMSO-d_6): δ = 2.36 (3H, s, $-CH_3$), 3.59 (2H, s, CH_2), 3.74 (3H, s, $-OCH_3$), 6.59-8.61 (6H, m, Ar-H), 10.62 (1H, s, =CH), 11.51 (1H, s, -NH, D_2O exchg.); 11.7 (1H, s, -CONH, D_2O exchg.); ^{13}C NMR (125 MHz, DMSO-d_6): δ = 11.5 (CH_3), 27.8 (CH_2), 55.0 (OCH_3), 100.2, 103.7, 109.4, 110.8, 127.8, 128.7, 129.2, 130.0, 133.4, 133.6, 134.0, 137.5, 140.9, 154.0, 168.0, 172.0S; MS: *m/z* = 390.26 $[M]^+$; Analysis: for $C_{19}H_{17}N_3O_2Cl_2$, calcd. C 58.47, H 4.35, N 10.77 %; found C 58.25, H 4.33, N 10.74 %.

2-(5-Methoxy-2-methyl-1H-indol-3-yl)-N′-[(E)-(3,4-dimethoxyphenyl)methylidene] acetohydrazide **(S7):** Yield: 70%; m.p.: 210–212°C; IR (KBr) cm^{-1}: 3299 (NH), 3011 (C-H), 1654 (C=O), 1599 (C=N); ^{1}H NMR (500 MHz, DMSO-d_6): δ = 2.40 (3H, s, $-CH_3$), 3.70 (2H, s, CH_2), 3.84 (3H, s, $-OCH_3$), 6.50-8.40 (6H, m, Ar-H), 10.50 (1H, s, =CH), 11.20 (1H, s, -NH, D_2O exchg.), 11.5 (1H, s, -CONH, D_2O exchg.); ^{13}C NMR (125 MHz, DMSO-d_6): δ = 12.1 (CH_3), 12.2 (CH_2), 55.6 (OCH_3), 55.8 (OCH_3), 56.0 (OCH_3), 101.0, 121.4, 122.0, 127.5, 143.1, 151.0, 175.0; MS: *m/z* = 381.42 $[M]^+$; Analysis: for $C_{21}H_{23}N_3O_4$, calcd. C 66.13, H 6.08, N 11.02 %; found C 66.31, H 6.10, N 11.05 %.

2-(5-Methoxy-2-methyl-1H-indol-3-yl)-N′-[(E)-(2-methoxyphenyl)methylidene] acetohydrazide **(S8)**: Yield: 60%; m.p.: 220–222°C; IR (KBr) cm^{-1}: 3315 (NH), 3017 (C-H), 1664 (C=O), 1601 (C=N); ^{1}H NMR (500 MHz, DMSO-d_6): δ = 2.36 (3H, s, $-CH_3$), 3.56 (2H, s, CH_2), 3.87 (3H, s, $-OCH_3$), 7.00-8.82 (11H, m, Ar-H), 10.61 (1H, s, =CH), 11.23(1H, s, NH, D_2O exchg.), 11.92 (1H, s, -CONH, D_2O exchg.); ^{13}C NMR (125 MHz, DMSO-d_6): δ = 12.1

(CH_3), 28.1 (CH_2), 56.1 (OCH_3), 63.4 (OCH_3), 111.2, 112.2, 112.3, 121.2, 122.7, 125.8, 126.0, 129.0, 130.0, 132.1, 137.0, 144.0, 158.2, 162.3; MS: m/z = 351.39 $[M]^+$; Analysis: for $C_{20}H_{21}N_3O_3$, calcd. C 68.36, H 6.02, N 11.96 %; found C 68.50, H 6.00, N 11.93 %.

N′-[(E)-(4-hydroxyphenyl)methylidene]-2-(5-methoxy-2-methyl-1H-indol-3-yl)acetohydrazide **(S9):** Yield: 70%; m.p.: 230–232 °C; IR (KBr) cm^{-1}: 3411 (OH), 3411 (NH), 3300 (C-H), 1654 (C=O), 1609 (C=N); ^1H NMR (500 MHz, DMSO-d_6): δ = 2.37 (3H, s, -CH_3), 3.58 (2H, s, CH_2), 3.75 (3H, s, -OCH_3), 6.59-8.36 (7H, m, Ar-H), 9.88 (1H, s, OH, D_2O exchg.), 10.60 (1H, s, =CH), 11.0 (1H, s, -CONH, D_2O exchg.), 11.73 (1H, s, -CONH, D_2O exchg.); ^{13}C NMR (125 MHz, DMSO-d_6): δ = 12.1 (CH_3), 28.2 (CH_2), 55.5 (OCH_3), 100.8, 104.9, 109.8, 110.0, 116.1, 128.9, 129.4, 130.5, 134.2, 143.4, 153.3, 159.5, 162.2, 167.4, 170.7, 172.9; MS: m/z = 337.37 $[M]^+$; Analysis: for $C_{19}H_{19}N_3O_3$, calcd. C 67.64, H 5.68, N 12.46 %; found C 67.43, H 5.70, N 12.43 %.

N′-[(E)-(3-hydroxyphenyl)methylidene]-2-(5-methoxy-2-methyl-1H-indol-3-yl)acetohydrazide (**S10**): Yield: 60%; m.p.: 145–147 °C; IR (KBr) cm^{-1}: 3500 (OH), 3413 (NH), 3023 (C-H), 1654 (C=O), 1617 (C=N); ^1H NMR (500 MHz, DMSO-d_6): δ = 2.37 (3H, s, -CH_3), 3.57 (2H, s, CH_2), 3.74 (3H, s, -OCH_3), 6.58-8.17 (7H, m, Ar-H), 9.59 (1H, s, OH, D_2O exchg.), 10.62 (1H, s, =CH), 11.21 (1H, s, -NH, D_2O exchg.), 11.39 (1H, s, -CONH, D_2O exchg.); ^{13}C NMR (125 MHz, DMSO-d_6): δ = 11.6 (CH_3), 27.6 (CH_2), 54.9 (OCH_3), 100.1, 104.0, 109.3, 110.8, 112.4, 117.2, 118.2, 128.5, 129.5, 130.0, 133.8, 135.5, 146.2, 152.8, 157.5, 175.0; MS: m/z = 337.37 [M]+; Analysis: for $C_{19}H_{19}N_3O_3$, calcd. C 67.64, H 5.68, N 12.46 %; found C 67.41, H 5.70, N 12.42 %.

2-(5-Methoxy-2-methyl-1H-indol-3-yl)-N′-{(E)-[4-(dimethylamino) phenyl]methylidene}acetohydrazide (**S11**): Yield: 65%; m.p.: 200–202 °C; IR (KBr) cm^{-1}: 3351 (NH), 2909 (C-H), 1654 (C=O), 1609 (C=N); ^1H NMR (500 MHz, DMSO-d_6): δ = 2.37 (3H, s, -CH_3), 3.00 (6H, s, 2×NCH_3) 3.59 (2H, s, CH_2), 3.74 (3H, s, -OCH_3), 6.59-8.32 (7H, m, Ar-H), 10.60 (1H, s, =CH), 10.97 (1H, s, -NH, D_2O exchg.), 11.62 (1H, s, -CONH, D_2O exchg.); ^{13}C NMR (125 MHz, DMSO-d_6): δ = 12.1 (CH_3), 28.0 (CH_2), 30.0 (NCH_3), 31.0 (NCH_3), 55.5 (OCH_3), 100.9, 105.0, 110.0, 111.2, 112.27, 112.3, 122.2, 128.4, 128.7, 128.9, 129.8, 130.5, 143.9, 151.7, 153.3, 172.7; ms: m/z = 364.44 $[M]^+$; Analysis: for $C_{21}H_{24}N_4O_2$, calcd. C 69.21, H 6.64, N 15.37 %; found C 69.37, H 6.66, N 15.33 %.

2-(5-methoxy-2-methyl-1H-indol-3-yl)-N′-[(E)-(3-methoxyphenyl)methylidene] acetohydrazide (**S12**): Yield: 65%; m.p.: 195–197 °C; IR (KBr) cm^{-1}: 3412 (NH), 3000 (C-H), 1654 (C=O), 1636 (C=N); ^1H NMR (500 MHz, DMSO-d_6): δ = 2.37 (3H, s, -CH_3), 3.58 (2H, s, CH_2), 3.80 (3H, s, -OCH_3), 6.59-8.44 (7H, m, Ar-H), 10.67 (1H, s, =CH), 11.28 (1H, s, -NH, D_2O exchg.), 11.46 (1H, s, -CONH, D_2O exchg.); ^{13}C NMR (125 MHz, DMSO-d_6): δ = 12.1 (CH_3), 28.3 (CH_2), 55.5 (OCH_3), 55.35 (OCH_3), 100.8, 104.5, 104.7, 109.8, 110.0, 111.6, 120.3, 129.3, 130.3, 134.3, 134.3, 136.2, 142.9, 146.5, 153.3, 159.9, 173.3; MS: m/z = 351.39 $[M]^+$; Analysis: for $C_{20}H_{21}N_3O_3$, calcd. C 68.36, H 6.02, N 11.96 %; found C 68.15, H 6.00, N 11.99 %.

N′-[(E)-(4-ethoxyphenyl)methylidene]-2-(5-methoxy-2-methyl-1H-indol-3-yl)acetohydrazide (**S13**): Yield: 75%; m.p.: 213–215 °C; IR (KBr) cm^{-1}: 3322 (NH), 3042 (C-H), 1654 (C=O), 1571 (C=N); ^1H NMR (500 MHz, DMSO-d_6): δ = 1.33 (3H, t, J = 7.5 Hz, CH_2CH_3), 2.37 (3H, s, -CH_3), 3.57 (2H, s, CH_2), 3.74 (3H, s, -OCH_3), 4.06 (2H, q, J = 7.5 Hz, OCH_2), 6.58-8.20 (7H, m, Ar-H), 10.61 (1H, s, =CH), 11.12 (1H, s, -NH, D_2O exchg.), 11.30 (1H, s, -CONH, D_2O exchg.); ^{13}C NMR (125 MHz, DMSO-d_6): δ = 12.2 (CH_3), 15.0 (CH_2), 28.2 (CH_3), 55.5 (OCH_3), 55.88 (OCH_2), 63.7, 100.8, 104.8, 109.8, 111.2, 115.1, 127.2, 129.2, 129.3, 130.5, 134.3, 143.0, 146.5, 153.3, 160.2, 167.5, 173.0; ms: m/z = 365.42 $[M]^+$; Analysis: for $C_{21}H_{23}N_3O_3$, calcd. C 69.02, H 6.34, N 11.50 %; found C 69.22, H 6.36, N 11.53 %.

2-(5-Methoxy-2-methyl-1H-indol-3-yl)-N′-[(E)-(2,4,5-trimethoxyphenyl) methylidene]acetohydrazide (**S14**): Yield: 60%; m.p.: 238–240 °C; IR (KBr) cm^{-1}: 3412 (NH), 2943 (C-H), 1654 (C=O), 1617 (C=N); ^1H NMR (500 MHz, DMSO-d_6): δ = 2.36 (3H, s, -CH_3), 3.51 (2H, s, CH_2), 3.78 (12H, s, -4×OCH_3), 6.91-8.42 (5H, m, Ar-H), 10.61 (1H, s, =CH), 11.14 (1H, s, -NH, D_2O exchg.), 11.42 (1H, s, -CONH, D_2O exchg.); ^{13}C NMR (125 MHz, DMSO-d_6): δ = 12.1 (CH_3), 30.1 (CH_2), 55.5 (OCH_3), 56.4 (OCH_3), 60.9 (OCH_3), 62.1 (OCH_3), 100.8, 104.8, 109.1, 109.8, 110.0, 111.2, 120.8, 130.5, 134.4, 138.9, 142.0, 152.8, 153.3, 155.2, 172.9; ms: m/z = 411.45 $[M]^+$; Analysis: for $C_{22}H_{25}N_3O_5$, calcd. C 64.22, H 6.12, N 10.21 %; found C 64.35, H 6.14, N 10.24 %.

2-(5-Methoxy-2-methyl-1H-indol-3-yl)-N′-[(E)-(2,3,4-trimethoxyphenyl) methylidene]acetohydrazide (**S15**): Yield: 55%; m.p.: 250–252 °C; IR (KBr) cm^{-1}: 3310 (NH), 3048 (C-H), 1654 (C=O), 1595 (C=N); ^1H NMR (500 MHz,

DMSO-d_6): δ = 2.37 (3H, s, -CH_3), 3.59 (2H, s, CH_2), 3.84 (12H, s, 4×-OCH_3), 6.59-8.74 (5H, m, Ar-H), 10.60 (1H, s, =CH), 11.08 (1H, s, -NH, D_2O exchg.), 11.33 (1H, s, -CONH, D_2O exchg.); ^{13}C NMR (125 MHz, DMSO-d_6): δ = 12.1 (CH_3), 28.5 (CH_2), 55.6 (OCH_3), 56.2 (OCH_3), 56.4 (OCH_3), 56.9 (OCH_3), 98.3, 101.1, 104.8, 108.4, 109.8, 111.2, 114.1, 129.2, 130.6, 134.4, 138.8, 143.6, 152.1, 153.6, 167.3, 172.9; ms: *m*/*z* = 411.45 [M]+; Analysis: for $C_{22}H_{25}N_3O_5$, calcd. C 64.22, H 6.12, N 10.21 %; found C 64.36, H 6.10, N 10.23 %.

2-(5-Methoxy-2-methyl-1H-indol-3-yl)-N′-[(E)-(3,4,5-trimethoxyphenyl) methylidene]acetohydrazide (**S16**): Yield: 58%; m.p.: 233–235 °C; IR (KBr) cm^{-1}: 3309 (NH), 3015 (C-H), 1654 (C=O), 1577 (C=N); ^1H NMR (500 MHz, DMSO-d_6): δ = 2.37 (3H, s, -CH_3), 3.59 (2H, s, CH_2), 3.83 (12H, s, 4× -OCH_3), 6.97-8.20 (5H, m, Ar-H), 10.61 (1H, s, =CH), 11.28 (1H, s, -NH, D_2O exchg.), 11.40 (1H, s, -CONH, D_2O exchg.); ^{13}C NMR (125 MHz, DMSO-d_6): δ = 12.1 (CH_3), 28.4 (CH_2), 55.6 (OCH_3), 55.8 (OCH_3), 56.3 (OCH_3), 60.5 (OCH_3), 60.5, 101.1, 104.4, 104.6, 104.7, 109.8, 111.2, 130.3, 130.6, 134.2, 139.3, 142.9, 153.3, 153.6, 173.2; ms: *m*/*z* = 411.45 [M]+; Analysis: for $C_{22}H_{25}N_3O_5$, calcd. C 64.22, H 6.12, N 10.21 %; found C 64.37, H 6.10, N 10.24 %.

2-(5-Methoxy-2-methyl-1H-indol-3-yl)-N′-[(E)-(2,4,6-trimethoxyphenyl) methylidene]acetohydrazide (**S17**): Yield: 55%; m.p.: 230–232 °C; IR (KBr) cm^{-1}: 3412 (NH), 3056 (C-H), 1654 (C=O), 1612 (C=N); ^1H NMR (500 MHz, DMSO-d_6): δ = 2.37 (3H, s, -CH_3), 3.59 (2H, s, CH_2), 3.82 (12H, s, 4× -OCH_3), 6.57-8.74 (5H, m, Ar-H), 10.61 (1H, s, =CH), 11.10 (1H, s, NH, D_2O exchg.), 11.80 (1H, s, -CONH, D_2O exchg.); ^{13}C NMR (125 MHz, DMSO-d_6): δ = 12.1 (CH_3), 28.4 (CH_2), 55.5 (OCH_3), 55.7 (OCH_3), 56.3 (OCH_3), 60.5 (OCH_3), 98.6, 106.7, 111.2, 115.6, 129.9, 142.2, 144.1, 153.3, 159.3, 159.6 162.1, 167.3, 172.9; ms: *m*/*z* = 411.45 [M]+; Analysis: for $C_{22}H_{25}N_3O_5$, calcd. C 64.22, H 6.12, N 10.21 %; found C 64.38, H 6.13, N 10.17 %.

2-(5-Methoxy-2-methyl-1H-indol-3-yl)-N′-[(E)-(2,4-dimethoxyphenyl) methylidene]acetohydrazide (**S18**): Yield: 60%; m.p.: 170–172 °C; IR (KBr) cm^{-1}: 3413 (NH), 3000 (C-H), 1654 (C=O), 1638 (C=N); ^1H NMR (500 MHz, DMSO-d_6): δ = 2.40 (3H, s, -CH_3), 3.60 (2H, s, CH_2), 3.82 (3H, s, -OCH_3), 6.50-8.70 (6H, m, Ar-H), 10.61 (1H, s, =CH), 11.20 (1H, s, NH, D_2O exchg.); 11.80 (1H, s, -CONH, D_2O exchg.); ^{13}C NMR (125 MHz, DMSO-d_6): δ = 11.5 (CH_3), 28.4 (CH_2), 55.0 (OCH_3), 55.6 (OCH_3), 55.68 (OCH_3), 98.2, 104.4, 106.2, 109.5, 110.7, 115.2, 128.8, 130.0, 132.0, 135.8, 138.3, 143.6, 152.8, 158.9, 162.0, 162.4, 164.7, 172.0; ms: *m*/*z* = 381.42 $[M]^+$; Analysis: for $C_{21}H_{23}N_3O_4$, calcd. C 66.13, H 6.08, N 11.02 %; found C 66.34, H 6.10, N 11.05 %.

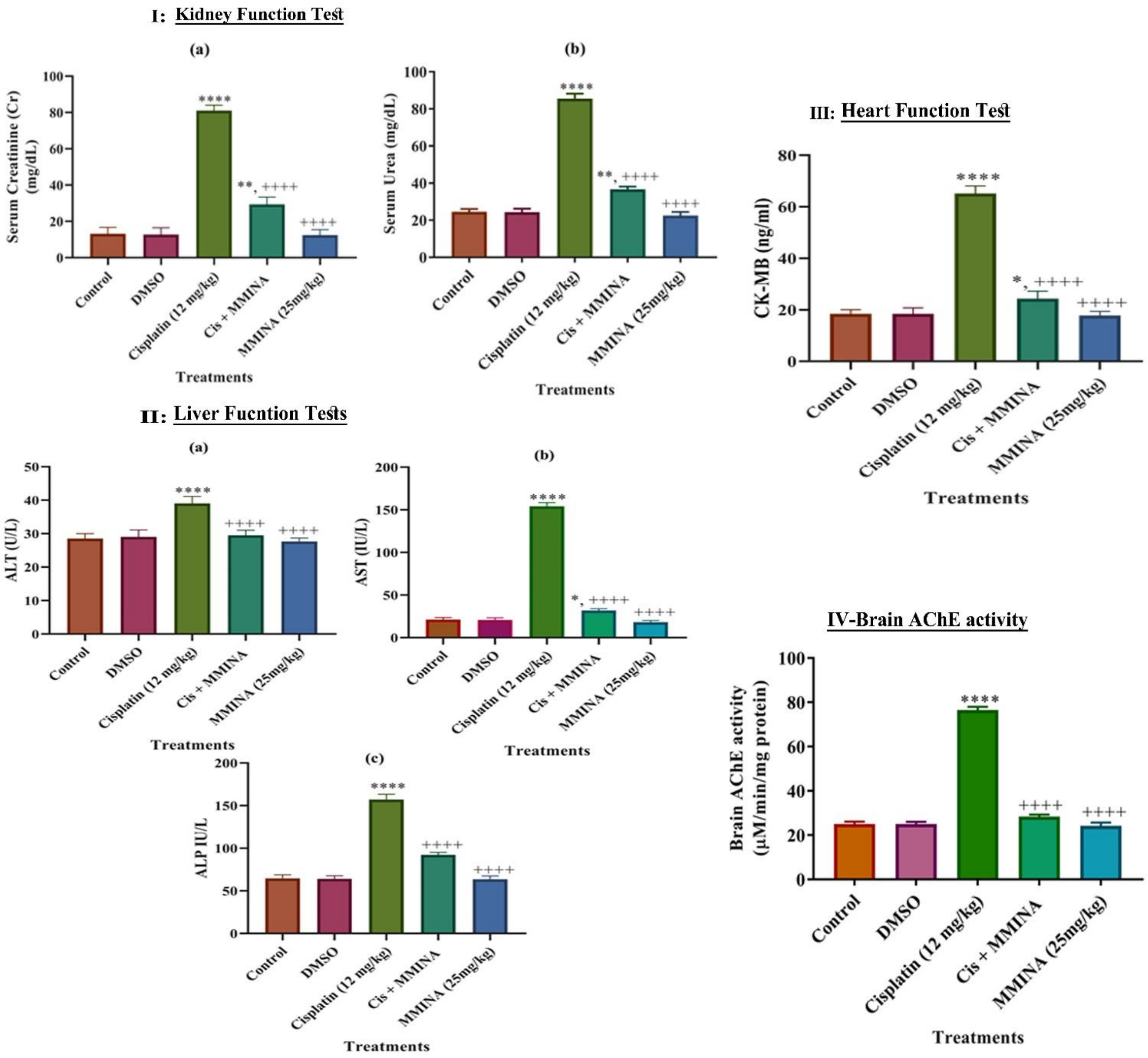

Supplementary Figure 2: Effects of MMINA on kidney, liver, heart and brain function marker enzymes in serum. Values are Mean ± SD (n= 6). I: kidney function test; (a) serum creatinine (mg/dl) levels and (b) serum urea content (mg/dl). II: Liver Function Test; (a) serum Alanine aminotransferase (ALT), Aspartate Aminotransferase (AST) and Alkaline phosphatase (ALP*). asterisks* *, **, **** indicate significance from the control group at $p < 0.05$, $p < 0.01$ and $p < 0.0001$ probability level, ++++ indicate significance from the Cisplatin group at $p < 0.0001$ probability level (One-way ANOVA followed by Tukey's multiple comparison tests).

Supplementary file S3:

Molecular docking, pharmacokinetic studies, and in vivo pharmacological study of indole derivative 2-(5-Methoxy-2-methyl-1H-indole-3-yl)-N'-[(E)-(3-nitrophenyl) methylidene] acetohydrazide as a promising chemoprotective agent against Cisplatin induced organ damage

Suhail Razak[1*,#], Tayyaba Afsar[1#], Nousheen Bibi[2#], Mahmoud Abulmeaty[1], Wajhul Qamar[3], Ali Almajwal[1], Anam Inam[2], Mashooq Ahmad Bhat[4], Dara Al Disi[1].

Western blot images

Kidney Tissue Uncropped Blots

COX2

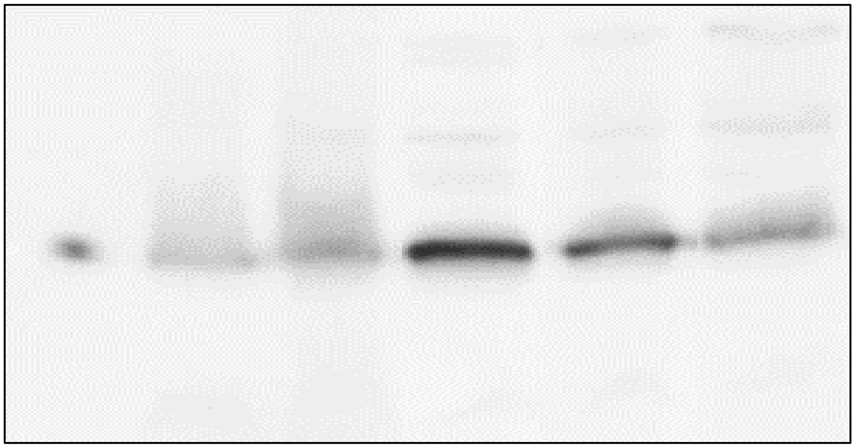

IL 1

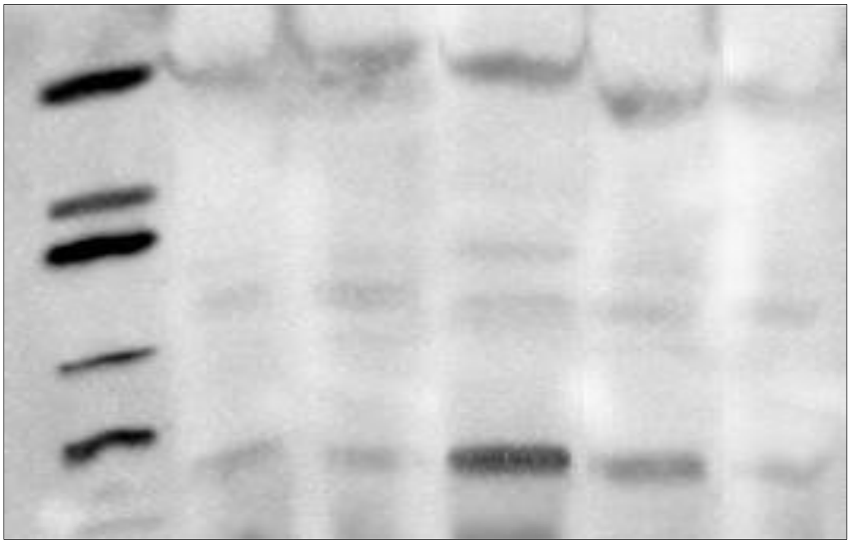

TNF-α

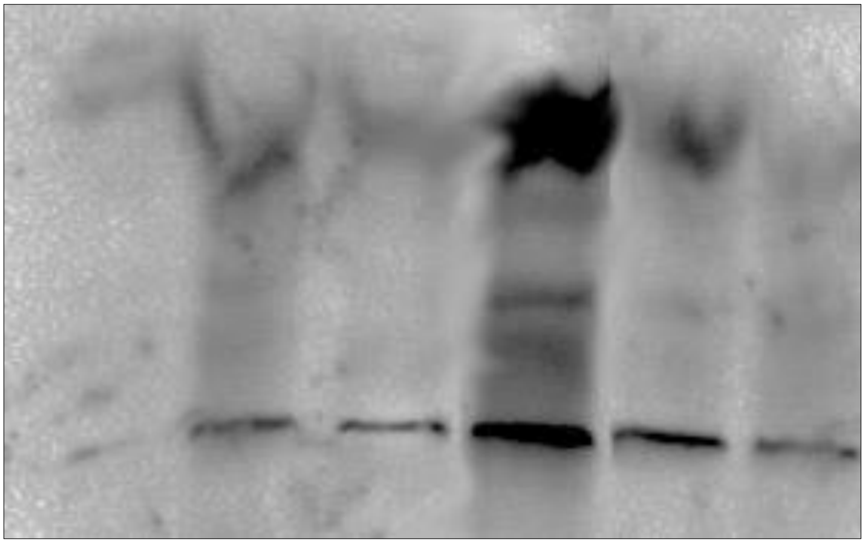

(i)

TNF-α

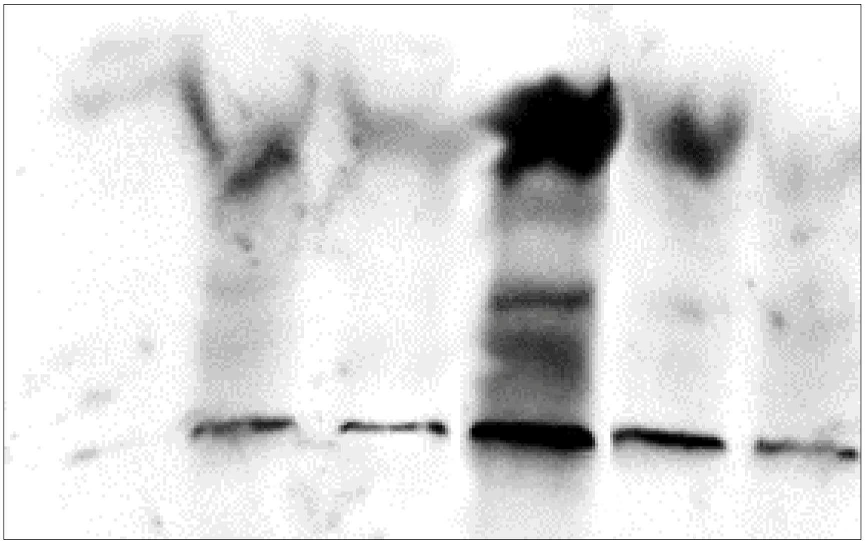

(ii)

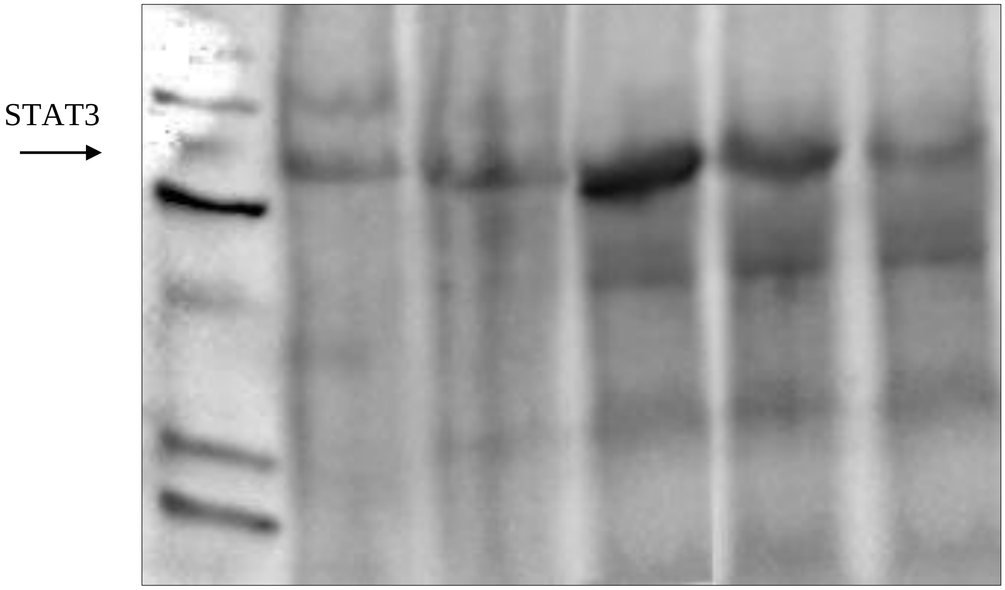

(i)

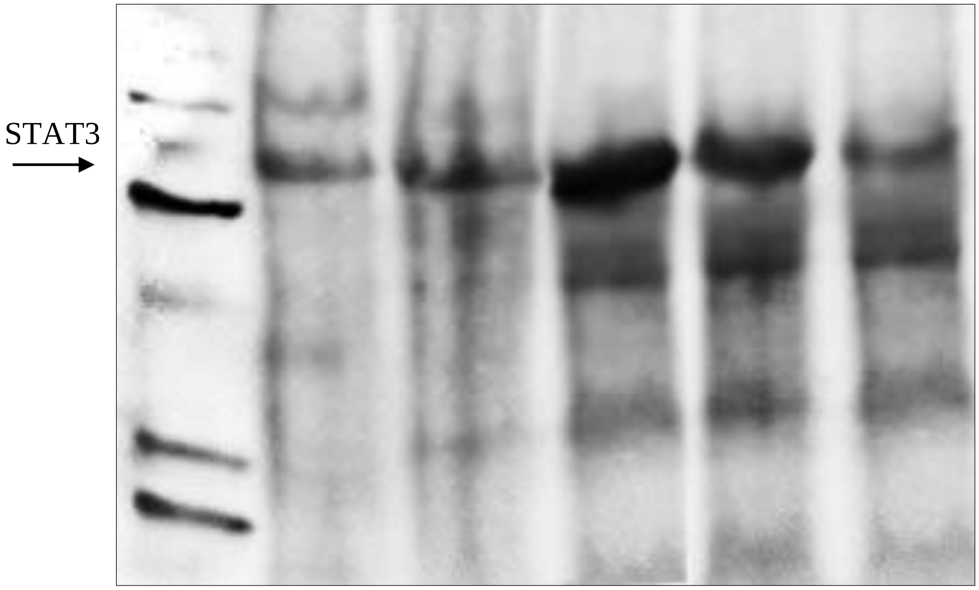

(ii)

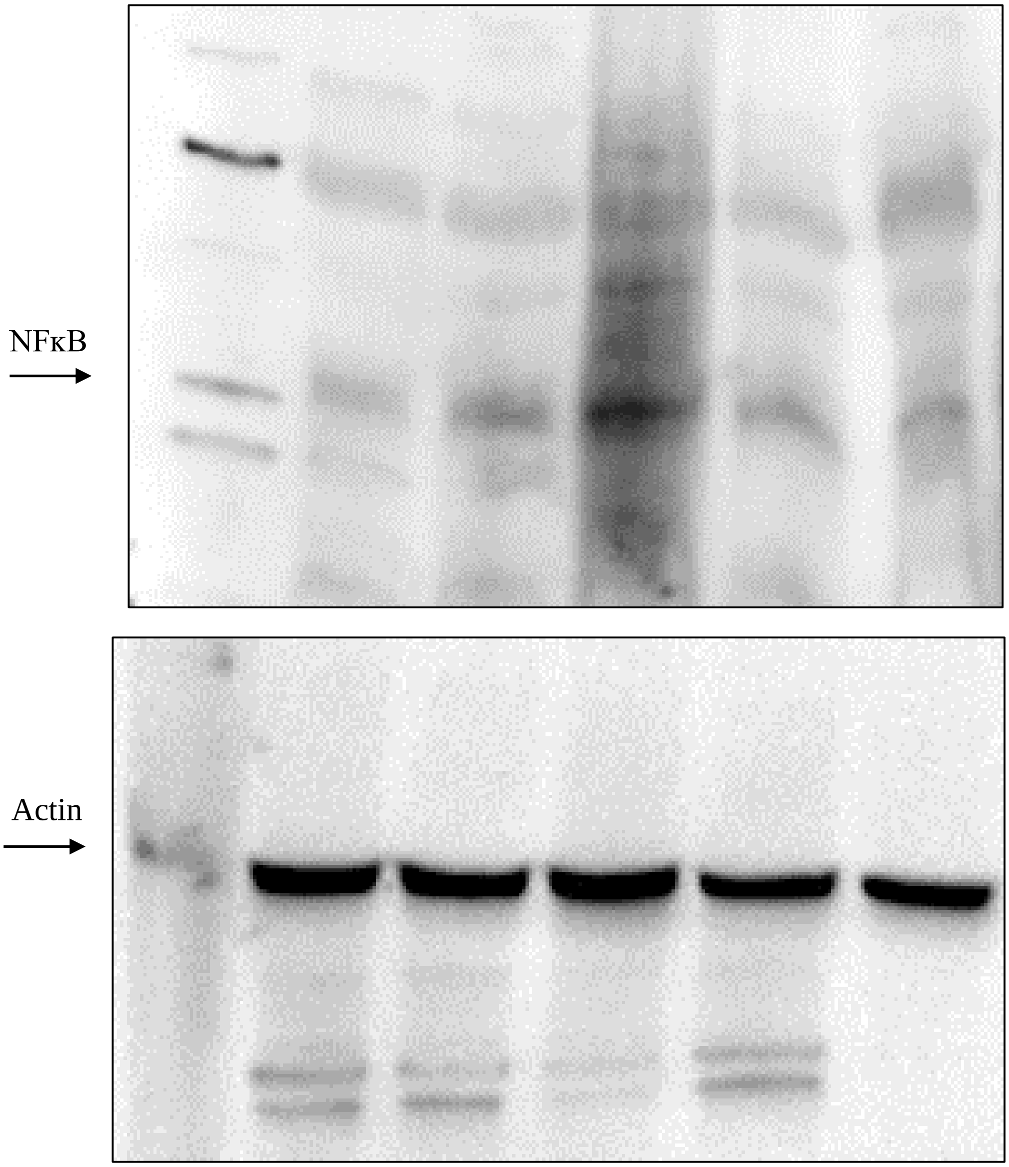
NFκB
Actin

Heart Tissue Uncropped Blots

STAT3

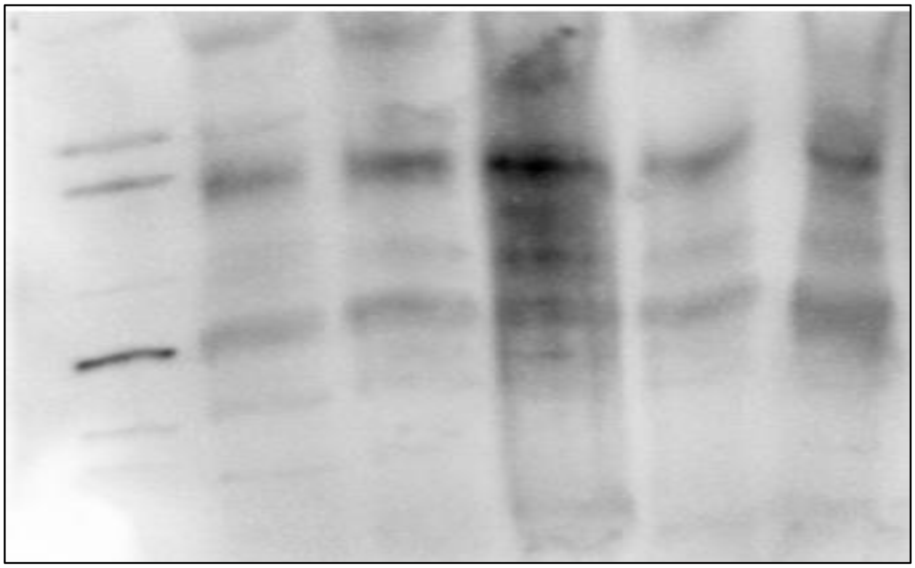

NFκB p65

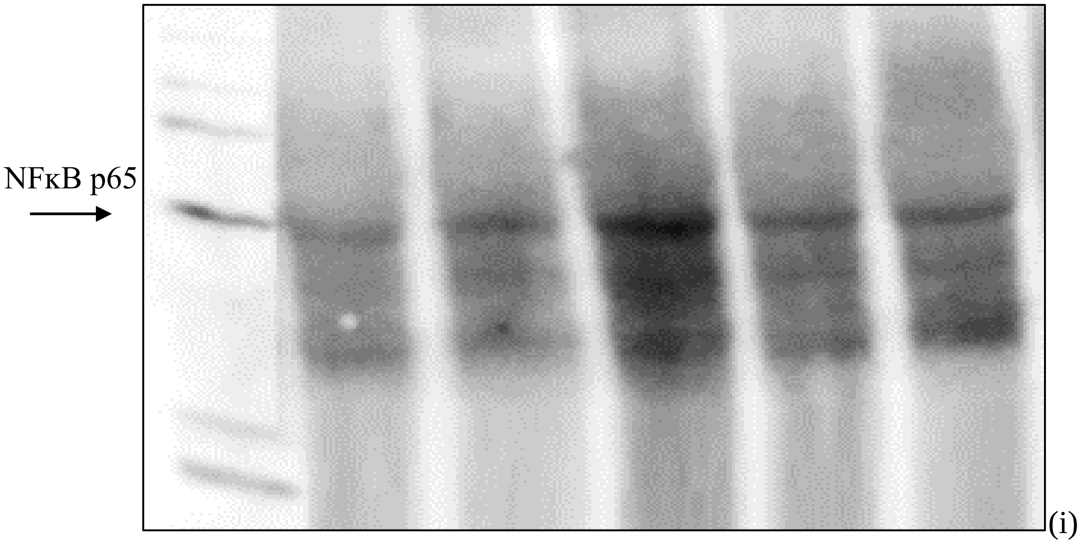

(i)

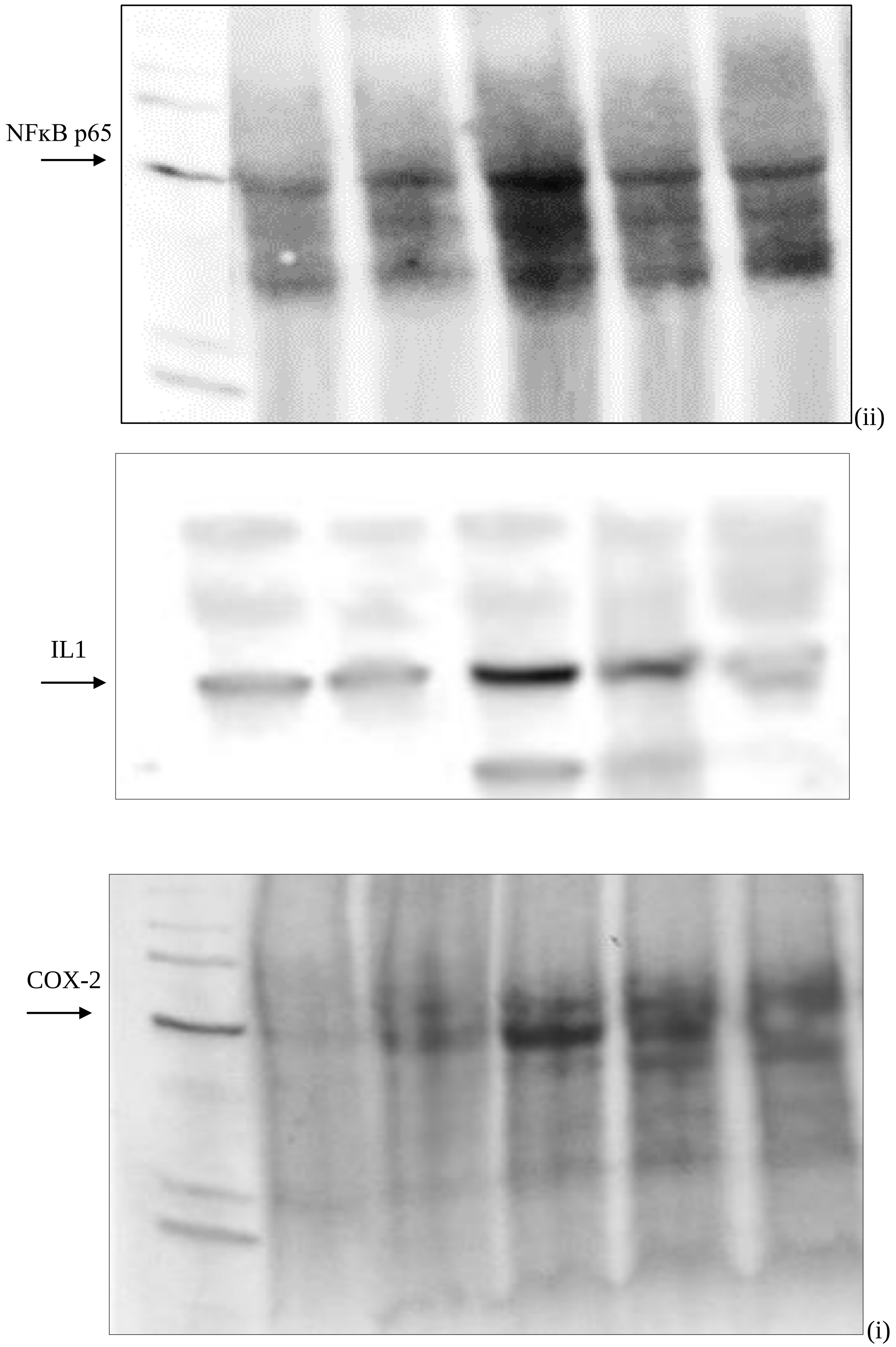

NFκB p65
(ii)
IL1
COX-2
(i)

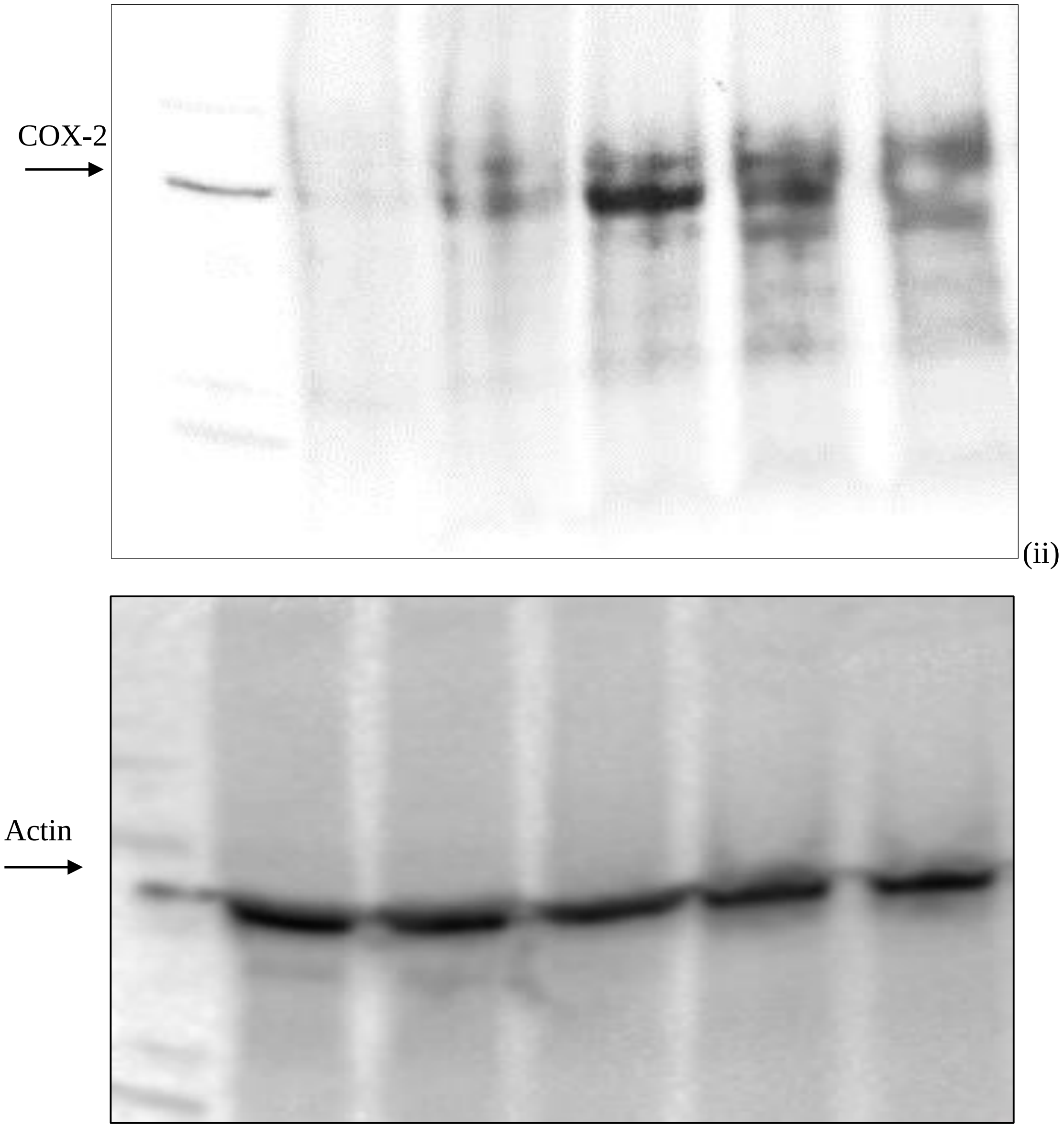
COX-2
(ii)
Actin

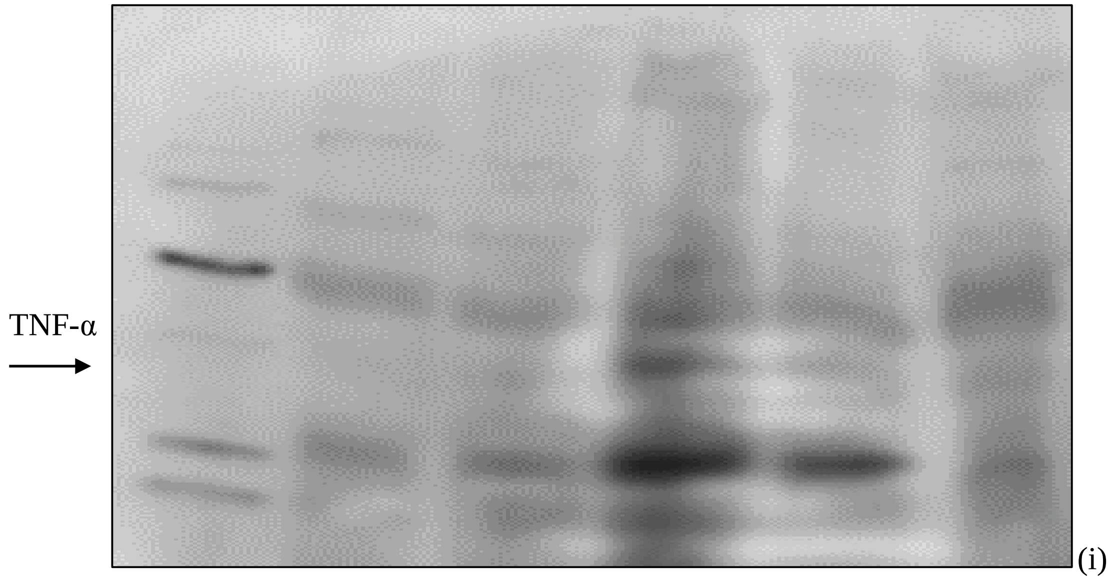
TNF-α
(i)

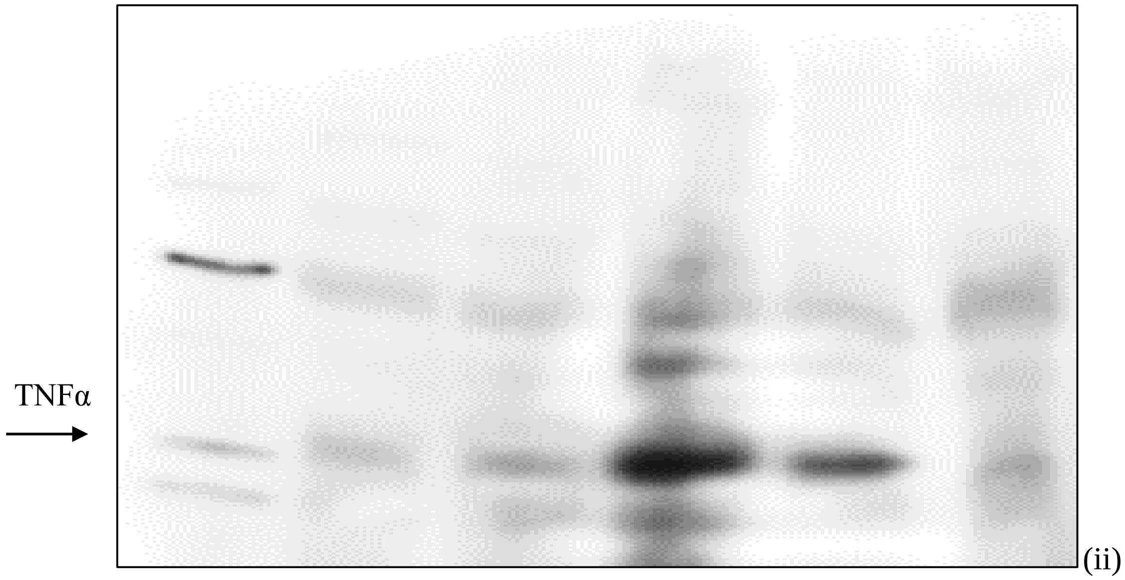
TNFα
(ii)

Liver tissue uncropped Blots

IL1

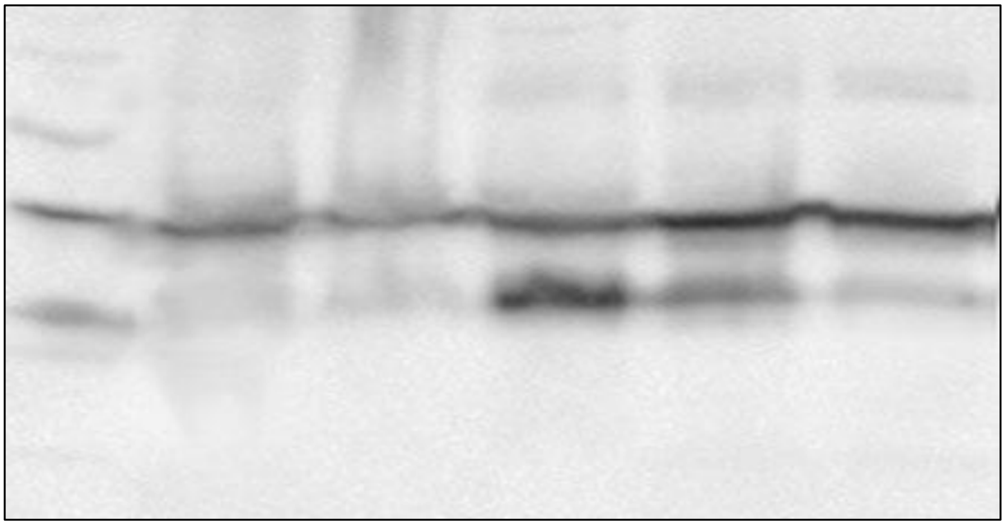

STAT 3

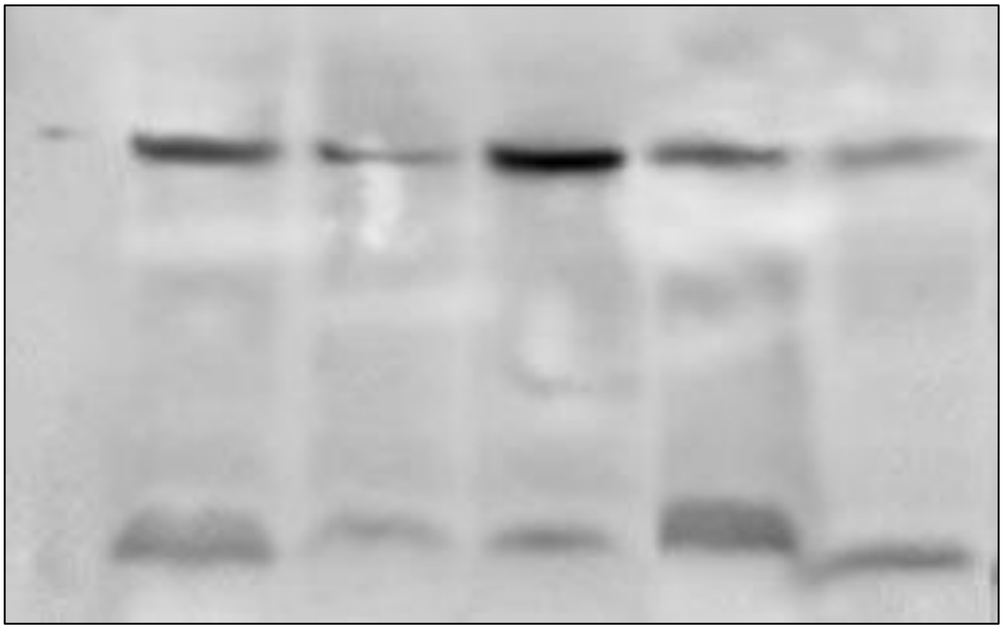

NFkB p65

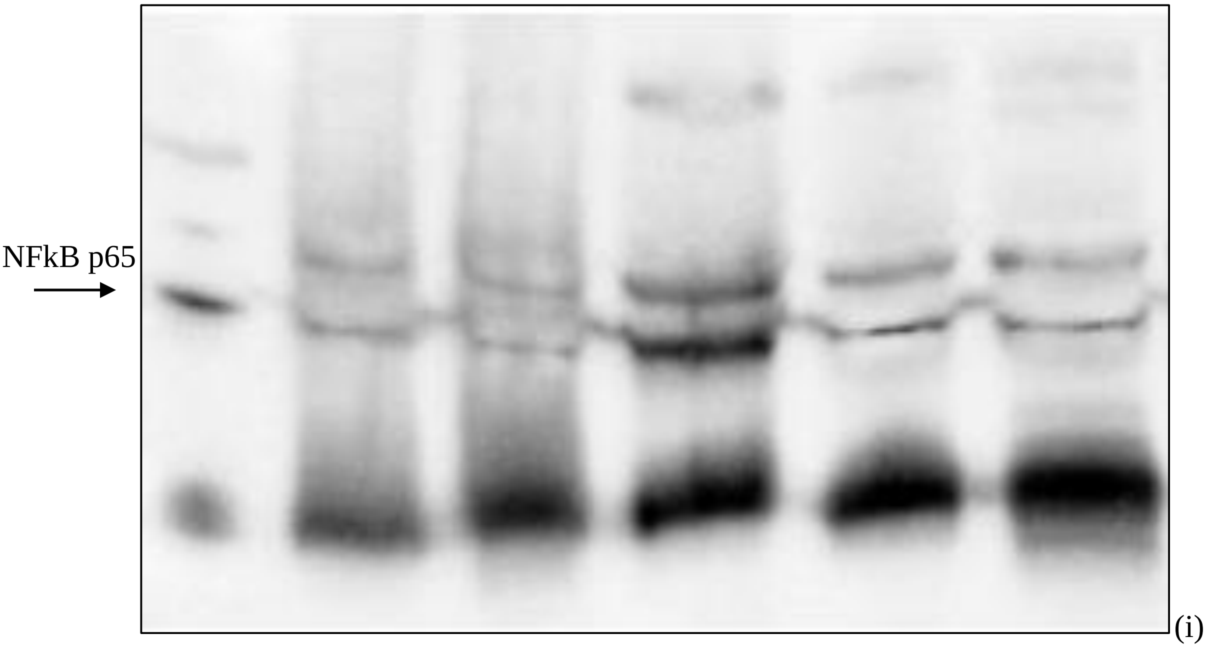

(i)

NFkB p65

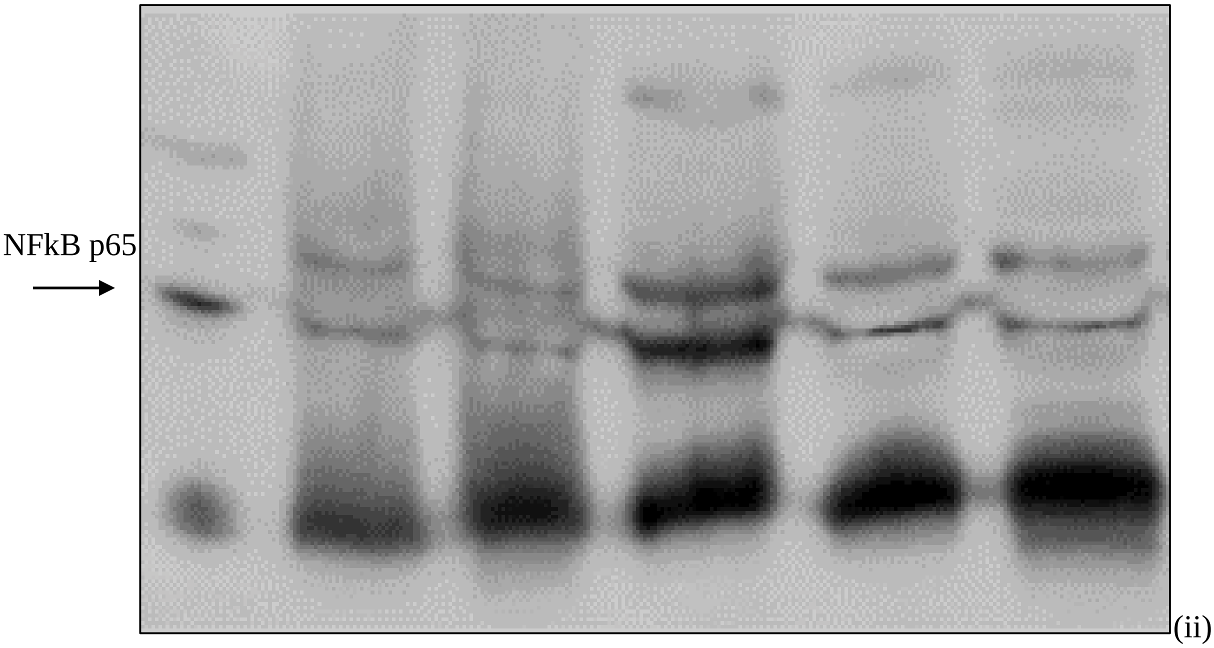

(ii)

COX-2

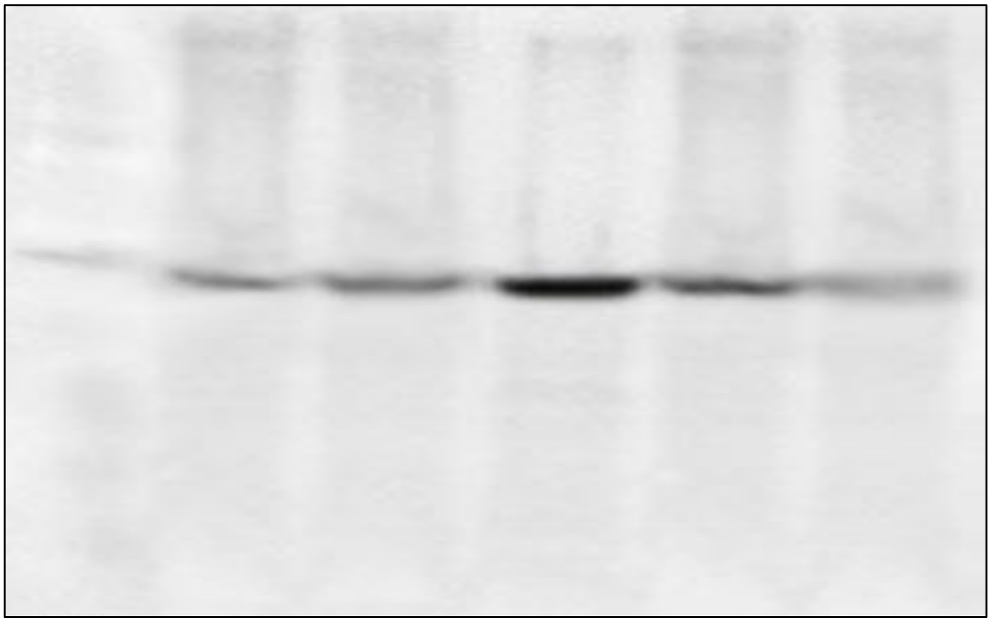

TNF-α

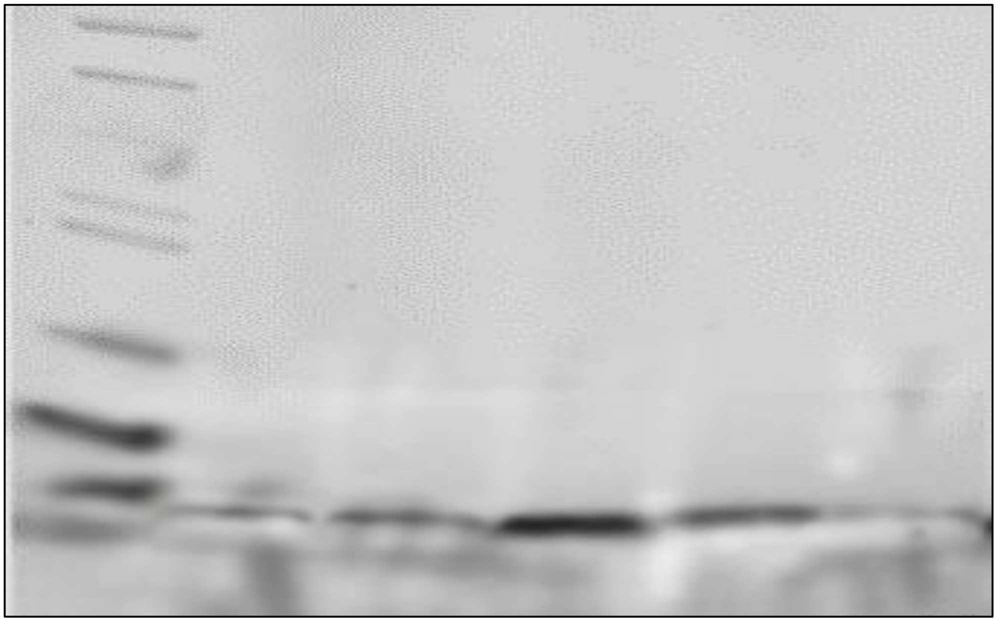

Actin

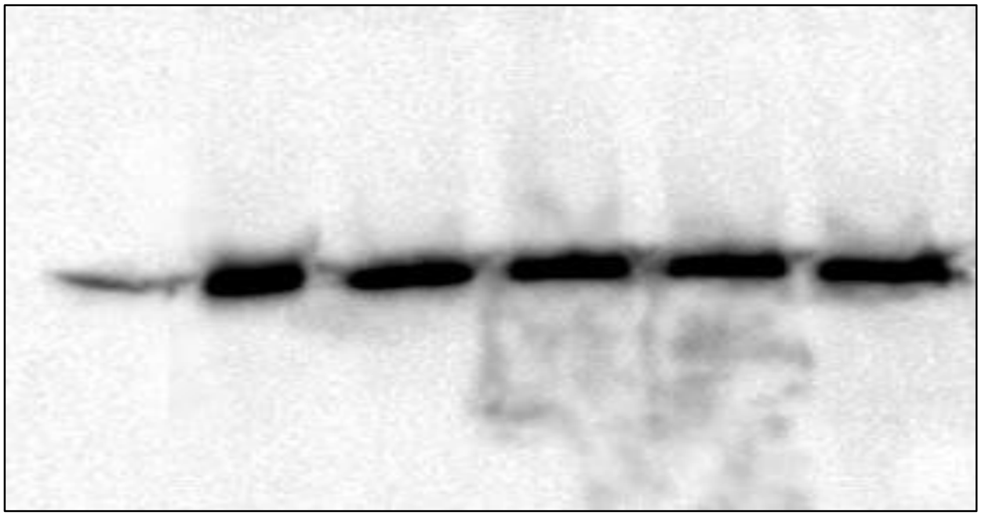

Brain tissue uncropped blots

IL 1

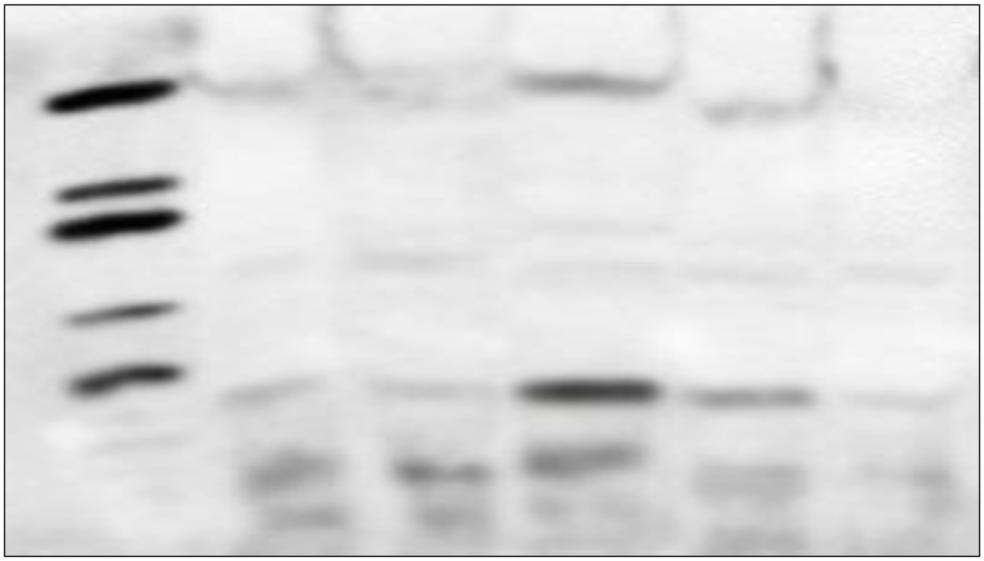

TNF-α

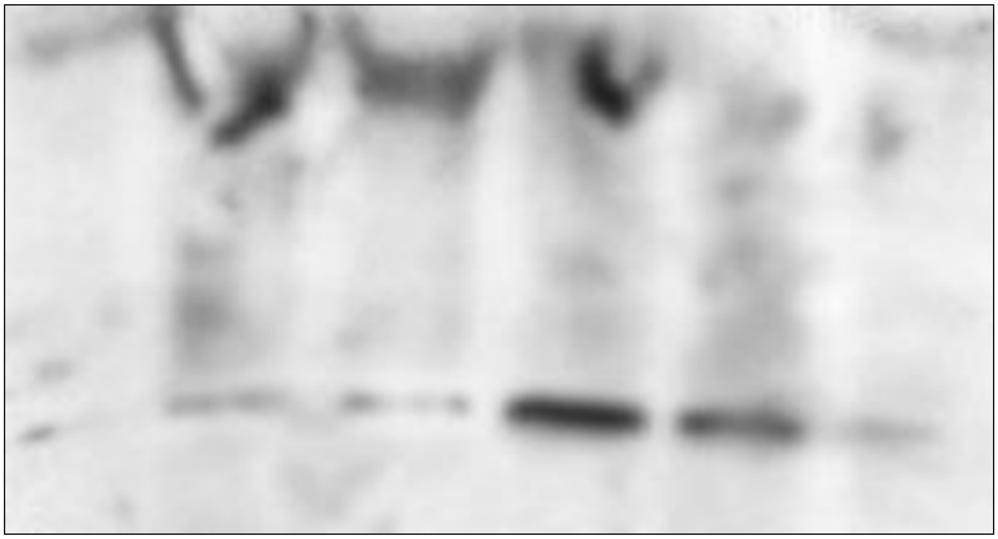

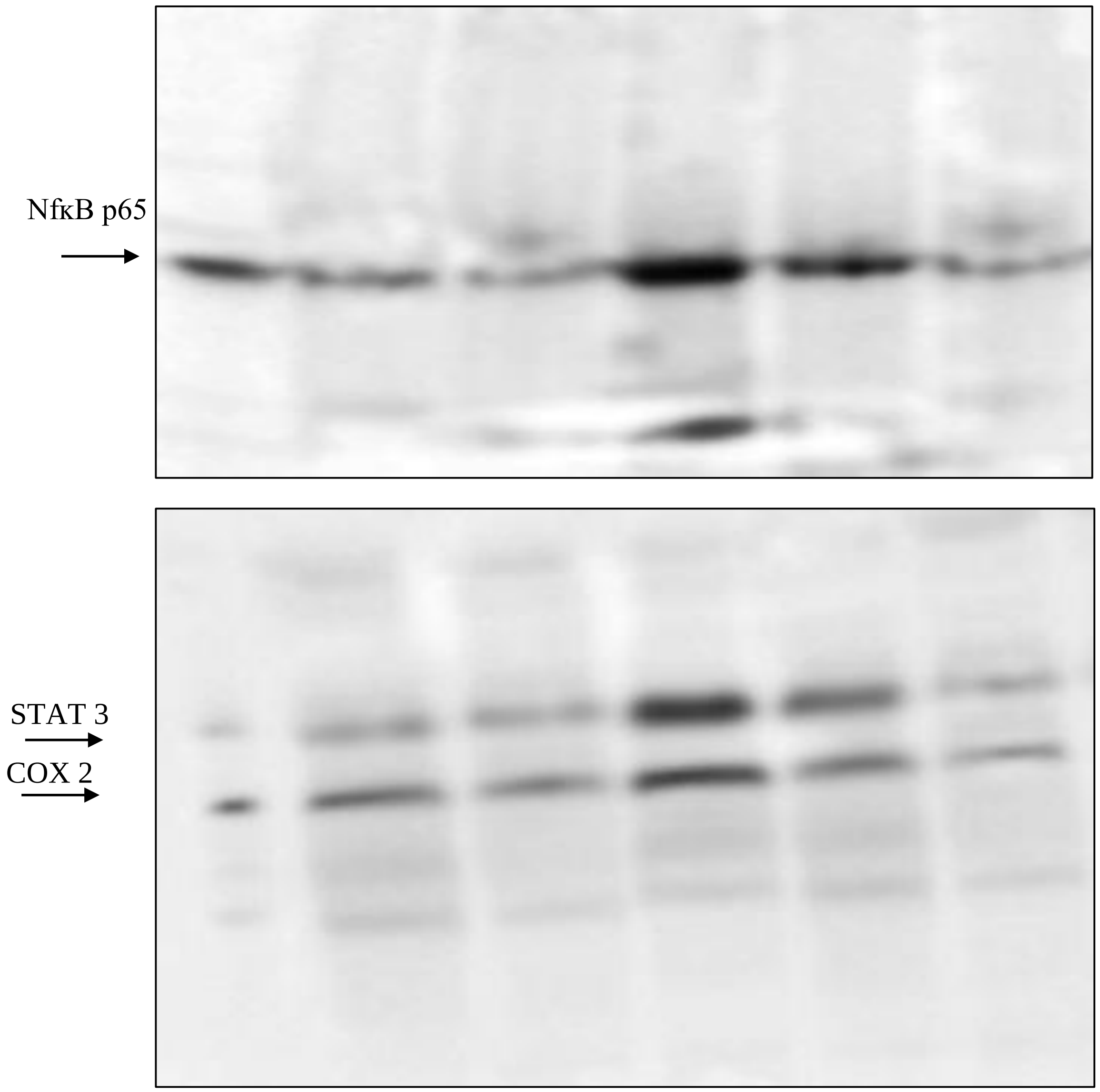

NfκB p65
STAT 3
COX 2

Actin

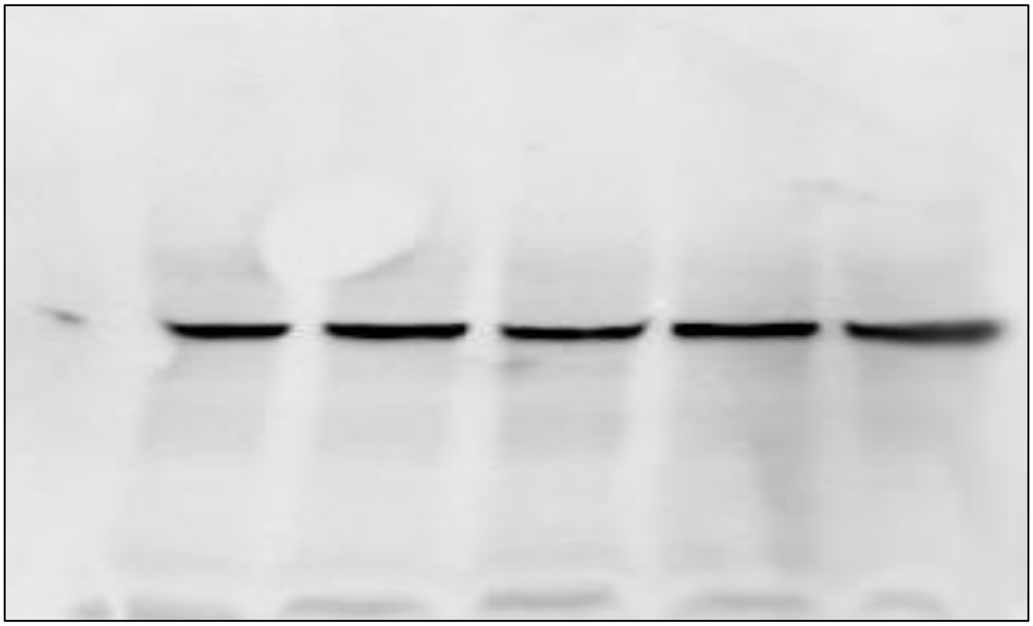

.

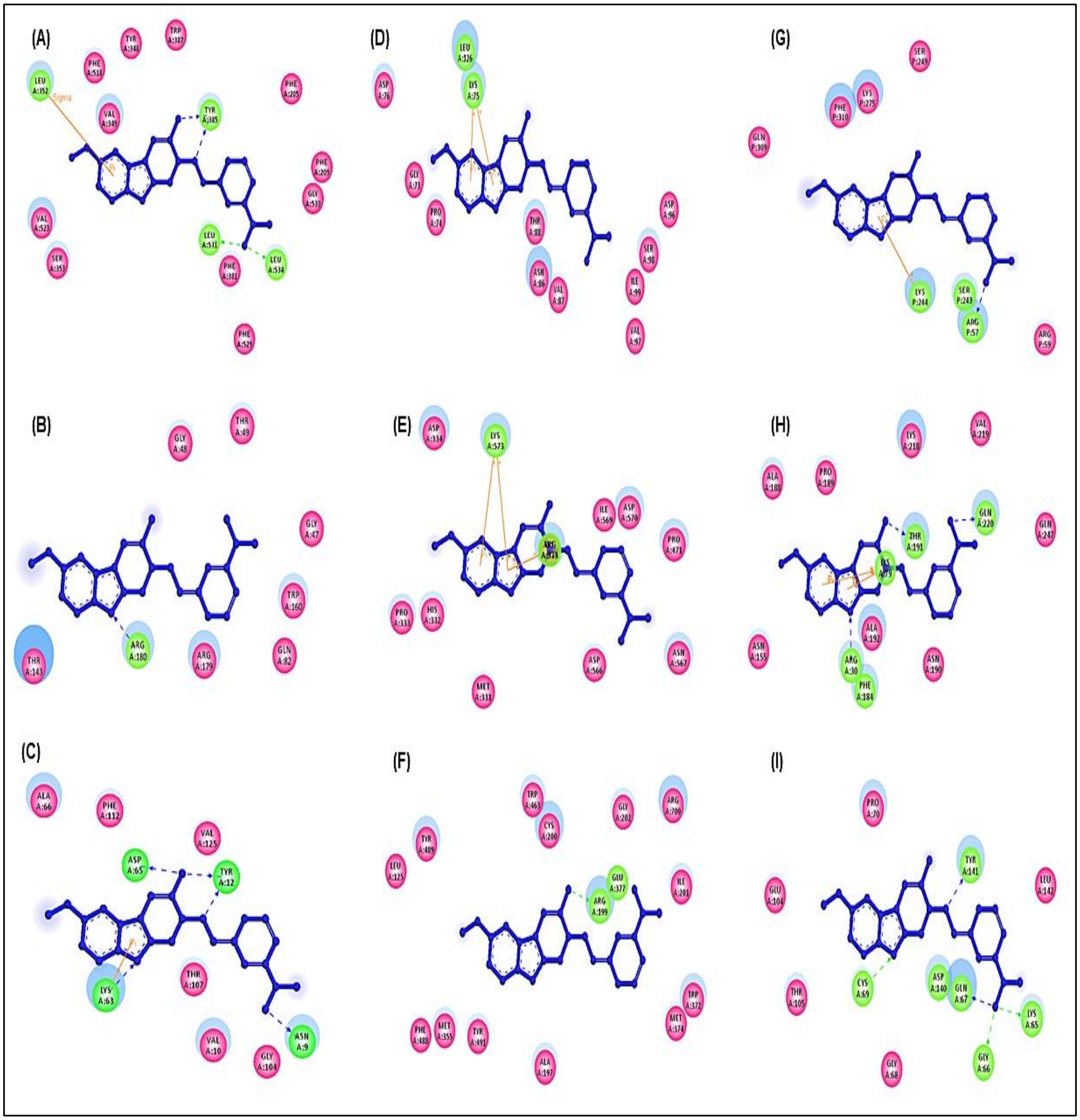

Supplementary Figure 3: 2D molecular docking interactions of all studied target molecules. (A) COX2 (B) GPx, (C) IL1 (D) SOD, (E) STAT3, (F) iNOS, (G) NFKB, (H) p65 and (I) TNF-α
